# Lycopene β-cyclase expression influences plant physiology, development, and metabolism in tobacco plants

**DOI:** 10.1093/jxb/erab029

**Published:** 2021-01-23

**Authors:** Stella Kössler, Tegan Armarego-Marriott, Danuše Tarkowská, Veronika Turečková, Shreya Agrawal, Jianing Mi, Leonardo Perez de Souza, Mark Aurel Schöttler, Anne Schadach, Anja Fröhlich, Ralph Bock, Salim Al-Babili, Stephanie Ruf, Arun Sampathkumar, Juan C Moreno

**Affiliations:** 1 Max Planck Institut für Molekulare Pflanzenphysiologie, Am Mühlenberg1 D-14476, Potsdam-Golm, Germany; 2 Laboratory of Growth Regulators, Institute of Experimental Botany, The Czech Academy of Sciences and Palacký University, Šlechtitelů, Olomouc, Czech Republic; 3 Center for Desert Agriculture, Biological and Environmental Science and Engineering Division (BESE), King Abdullah University of Science and Technology (KAUST), Thuwal, Saudi Arabia; 4 Lancaster University, UK

**Keywords:** β-Carotene, biomass, carotenoids, lycopene β-cyclase, *Nicotiana tabacum* cv. Petit Havana, photosynthesis, phytohormones, RNAi, transplastomic

## Abstract

Carotenoids are important isoprenoids produced in the plastids of photosynthetic organisms that play key roles in photoprotection and antioxidative processes. β-Carotene is generated from lycopene by lycopene β-cyclase (LCYB). Previously, we demonstrated that the introduction of the *Daucus carota* (carrot) *DcLCYB1* gene into tobacco (cv. Xanthi) resulted in increased levels of abscisic acid (ABA) and especially gibberellins (GAs), resulting in increased plant yield. In order to understand this phenomenon prior to exporting this genetic strategy to crops, we generated tobacco (*Nicotiana tabacum* cv. Petit Havana) mutants that exhibited a wide range of *LCYB* expression. Transplastomic plants expressing *DcLCYB1* at high levels showed a wild-type-like growth, even though their pigment content was increased and their leaf GA_1_ content was reduced. RNA interference (RNAi) *NtLCYB* lines showed different reductions in *NtLCYB* transcript abundance, correlating with reduced pigment content and plant variegation. Photosynthesis (leaf absorptance, *F*_v_/*F*_m_, and light-saturated capacity of linear electron transport) and plant growth were impaired. Remarkably, drastic changes in phytohormone content also occurred in the RNAi lines. However, external application of phytohormones was not sufficient to rescue these phenotypes, suggesting that altered photosynthetic efficiency might be another important factor explaining their reduced biomass. These results show that *LCYB* expression influences plant biomass by different mechanisms and suggests thresholds for *LCYB* expression levels that might be beneficial or detrimental for plant growth.

## Teaser

A lycopene β-cyclase expression threshold influences plant biomass, development, and metabolism by modulating pigment and phytohormone content in transplastomic and RNAi tobacco lines.

## Introduction

Carotenoids are 40-carbon isoprenoids with polyene chains containing up to 15 conjugated double bonds (Hirschberg, 2001). In plants, carotenoids have many important functions, such as chlorophyll protection from UV light, and multiple antioxidant activities, including the ability to scavenge reactive oxygen species (ROS), such as singlet oxygen, and peroxyl radicals (Krinsky, 1989). In addition, carotenoids play a critical role in light absorption processes such as photosynthetic light harvesting via singlet state energy transfer (Frank and Cogdell, 1993; Dall’Osto *et al.*, 2007*b*; Andrade-Souza *et al.*, 2011). As membrane-bound compounds, they provide membrane stabilization (Frank and Cogdell, 1993, 1996; Demmig-Adams *et al.*, 1996; Havaux, 1998). Furthermore, carotenoids are structural constituents of the photosynthetic machinery, forming pigment–protein complexes in photosystems (PS) I and II and their respective light harvesting complexes (LHCs) as well as in the cytochrome *b*_6_*f* complex (Dall’Osto *et al.*, 2007*b*, 2014). Moreover, they are precursors of the apocarotenoid phytohormone abscisic acid (ABA), which is involved in developmental processes and adaptive stress responses to environmental stimuli such as drought-induced stomatal closure in plants (Nambara and Marion-Poll, 2005; Taylor *et al.*, 2005; Shi *et al.*, 2015), and of strigolactones (SLs), involved in plant development (Brewer *et al.*, 2013; Al-Babili and Bouwmeester, 2015). One of the most important carotenoids, for human health and nutrition is β-carotene, the precursor of vitamin A (Olson, 1996). β-Carotene deficiency leads to blindness, xerophthalmia, and premature death in humans (Giuliano *et al.*, 2003).

Lycopene-β-cyclase (LCYB) catalyses the last step of β-carotene synthesis, and therefore plays a key role in this pathway. Overexpression of *LCYB* leads to increased tolerance to abiotic stresses (e.g. drought and salt) in tomato (D’Ambrosio *et al.*, 2004), Arabidopsis (Chen *et al.*, 2011), and sweet potato (Kang *et al.*, 2018). Additionally, transgenic tomatoes (*Solanum lycopersicum*) expressing the tobacco *LCYB* gene showed tolerance to the bleaching herbicide 2-(4-chlorophenylthio) triethylamine (CPTA), a β-cyclase inhibitor (Ralley *et al.*, 2016). Moreover, increased β-carotene, violaxanthin, lutein, and zeaxanthin content in plants was shown to improve tolerance to abiotic stresses such as high light, UV irradiation, and salt stress by scavenging ROS (Davison *et al.*, 2002; Götz *et al*., 2002; Han *et al.*, 2008; Shi *et al.*, 2015; Kang *et al.*, 2018). In tobacco, overexpression of *NtLCYB* led to increased expression of genes for phytoene synthase (*PSY*), phytoene desaturase (*PDS*), ζ-carotene desaturase (*ZDS*), zeaxanthin epoxidase (*ZEP*), violaxanthin de-epoxidase (*VDE*), and neoxanthin synthase (*NXS*), increased accumulation of β-carotene, violaxanthin, lutein, and neoxanthin, and enhanced tolerance to salt and drought stress, whereas transgenic *NtLCYB* RNA interference (RNAi) lines either showed an albino phenotype in leaves or did not survive beyond the early developmental stages (Shi *et al.*, 2015).

**Fig. 1.**
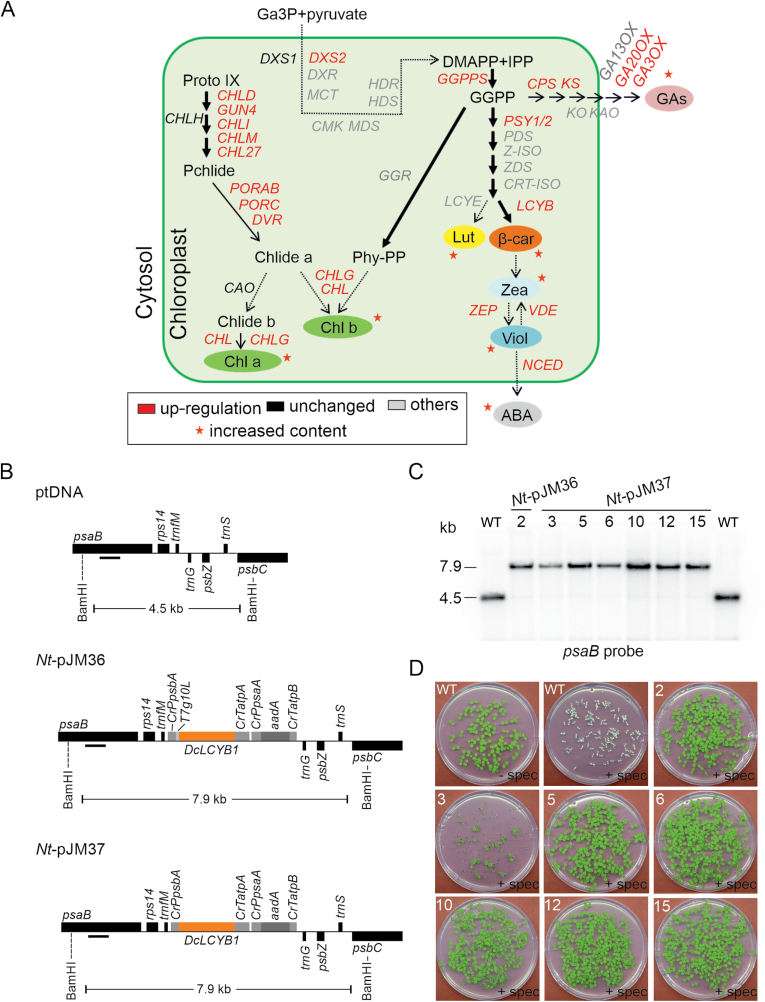
Introduction of the *Daucus carota* lycopene β-cyclase1 (*DcLCYB1*) gene into the plastid genome by stable transformation. (A) Schematic representation for isoprenoid-derived pathways (e.g. carotenoids, GA, ABA) of the changes caused upon expression of *DcLCYB1* in nuclear tobacco lines. Up-regulated genes are marked in red; genes shown in black were not changed; and genes shown in grey were not measured. Red stars indicate increased metabolite content. ABA, abscisic acid; β-car, β-carotene; Chl, chlorophyll; DMAPP, dimethylallyl diphosphate; GA, gibberellins; IPP, isopentenyl diphosphate; Lut, lutein; Pchlide, protochlorophillidae; Phy-PP, phytyl diphosphate; Viol, violaxanthin; Zea, zeaxanthin. Gene abbreviation, protein name, and a brief description of each gene measured by qPCR can be found in Moreno *et al.* (2020). (B) Physical map of the plastid genome region (ptDNA) used as a site for integration, and of the transgenic loci in the generated transplastomic tobacco lines (*Nt*-pJM37, *Nt*-pJM36). Native plastid genes are shown in black; introduced elements are shown in pale grey and orange. Genes above the line are transcribed from left to right; genes below the line are transcribed right to left. Promoter (P) and terminator (T) sequence from *Chlamydomonas reinhardtii* (*Cr*) are shown in pale grey. T7g10L is the gene 10 leader sequence from the T7 phage, which has been shown to be highly efficient at facilitating high-level translation in green plastids (Oey *et al.*, 2009; Elghabi *et al.*, 2011). The *aadA* gene encodes the AMINOGLYCOSIDE 3′-ADENYLYLTRANSFERASE enzyme, which confers resistance to the aminoglycoside-type antibiotics spectinomycin and streptomycin and as such serves as a selectable marker for transformed chloroplasts. The black box under *psaB* indicates the probe used for RFLP analysis. (C) RFLP analysis of transplastomic plants. Total DNA from wild-type and transformed lines was digested with *Bam*HI producing fragments of the sizes indicated in (A). A single, independently isolated transplastomic line is shown for *Nt*-pJM36; six lines are shown for *Nt*-pJM37. (D) Inheritance assay of transplastomic *DcLCYB1* plants. Germination of the T_1_ generation of plastid transformants in the presence of spectinomycin (+spec) revealed the homoplasmic stage for the integrated selectable marker gene *aadA*. Numbering of the lines refers to the nomenclature use in (B). spec, spectinomycin; WT, wild type.

Recently, a major growth advantage was reported in tobacco lines expressing the carrot *DcLCYB1* (Moreno *et al.*, 2016). This was shown under fully controlled (constant and fluctuating light regimes) and non-controlled climate conditions (Moreno *et al.*, 2020). Increased levels of photoprotective molecules such as β-carotene, lutein/zeaxanthin, and violaxanthin enhanced photoprotection and contributed to the increased photosynthetic efficiency, especially under fluctuating light conditions. However, unexpectedly, transgenic *DcLCYB1* lines also showed increased transcript levels of key genes involved in several metabolic pathways related to isoprenoid metabolism, resulting in increased biosynthesis of ABA and especially of different gibberellins (GAs; Fig. 1A). The increased GA/ABA ratio altered plant development and architecture, for example reducing shading of mature leaves. Thereby, induction of leaf senescence was delayed, and mature leaves maintained a high photosynthetic capacity. This ultimately resulted in higher fitness and yield in these tobacco lines (Moreno *et al.*, 2020).

The unexpected contribution of altered GA and ABA levels to the growth phenotype raises the question of whether or not similar effects have been overlooked so far in other mutants with altered carotenoid metabolism (Qin *et al.*, 2007; Li *et al.*, 2009; Cazzaniga *et al.*, 2012; Avendaño-Vázquez *et al*., 2014; Kromdijk *et al.*, 2016). For example, impaired growth of mutants with reduced carotenoid accumulation due to repression or inactivation of enzymes involved in carotenoid biosynthesis (Pogson *et al.*, 1998; Dall’Osto *et al.*, 2007*a*, 2013; Qin *et al.*, 2007; Li *et al.*, 2009; Cazzaniga *et al.*, 2012; Fiore *et al.*, 2012) has been solely attributed to defects in photosynthesis and impaired protection against oxidative stress, in line with the well-established functions of carotenoids. Possible additive effects of altered carotenoid biosynthesis on the synthesis of other isoprenoids, especially phytohormones, have not been considered so far. Therefore, a broader analysis is needed of the consequences of altered carotenoid biosynthesis for phytohormones, especially GAs, ABA, and strigolactones, whose synthesis is closely related to carotenoid synthesis.

In light of the reported growth benefit in tobacco (Moreno *et al.*, 2020), it is of interest to understand how a single gene transformation can trigger molecular and physiological responses that are reflected in higher yield. The higher *DcLCYB1* expression in the nuclear tobacco lines was reflected in a 2-fold increase in β-carotene content. In addition, higher *DcLCYB1* expression triggered a co-expression of key genes in carotenoid- and carotenoid-related pathways, which resulted, for instance, in higher chlorophyll and GA content, two important isoprenoid pathways influencing plant growth and development. Higher β-carotene was reflected in higher xanthophyll production, and therefore enhanced photoprotection, and ABA content. These lines of evidence suggest that *DcLCYB1* expression can induce various molecular pathways (Fig. 1A), at genetic and metabolic levels, that stimulate plant growth, development, photosynthesis, and yield. These findings bring into question the role of *LCYB* expression levels in growth regulation. Following this idea, higher *LCYB* expression levels might induce (stronger induction than in the nuclear *DcLCYB1* lines) the expression of key genes of carotenoid, ABA, GA, and chlorophyll biosynthesis pathways (Fig. 1A), and therefore, higher increases (compared with the nuclear lines) in β-carotene, ABA, GA, and chlorophyll contents. By contrast, a reduction in *LCYB* expression should trigger a down-regulation of key genes of carotenoid, ABA, GA, and chlorophyll biosynthesis pathways (Fig. 1A), and therefore, reductions in β-carotene, ABA, GA, and chlorophyll contents. In both cases, higher or reduced *LCYB* expression should lead to enhanced or reduced plant biomass, respectively. Based on the previous evidence we combined both cases and formulate the following hypothesis: *LCYB* expression regulates plant growth by influencing carotenoid (β-carotene and xanthophylls) and hormone (ABA and GA) contents through an activation of key genes of carotenoid and hormone biosynthesis pathways.

To test our hypothesis, we designed two genetic strategies to further increase (more than in the nuclear lines) and reduce *LCYB* expression in tobacco plants. One possible way to achieve very high transgene expression is via chloroplast transformation. Transformation of plastid DNA has enormous advantages over nuclear transformation (Bock, 2007; Clarke and Daniell, 2011). For instance, high transgene expression levels of more than 70% of soluble leaf protein can be achieved due to the large chloroplast number per cell, high ploidy per chloroplast, and high translation capacity of this compartment (Oey *et al.*, 2009). While expression levels of carotenoid biosynthesis enzymes in the range of several percent of soluble leaf protein is certainly not required to manipulate carotenoid biosynthesis, the appropriate choice of expression elements for chloroplast transformation allows the generation of mutants covering a wide range of transgene expression levels. As another advantage of chloroplast transformation, gene dispersal in the environment is largely abolished due to maternal inheritance of chloroplasts, which excludes plastid genes and transgenes from pollen transmission (Ruf *et al.*, 2007). Finally, transgene insertion via the homologous recombination process avoids position effects and gene silencing of the transgene, making plastid transformation ideal for crop improvement and metabolic engineering. Previously, plastid transformation approaches aiming to increase isoprenoid content succeeded in tobacco and tomato (Apel and Bock, 2009; Kumar *et al.*, 2012; Lu *et al.*, 2013). Tomato lines expressing the daffodil (*Narcissus pseudonarcissus*) *LCYB* gene from the plastid genome (transplastomic lines) showed increased β-carotene content in the fruit (changed from red to orange), but not in the leaves (Apel and Bock, 2009). In addition, β-carotene content measured in leaves of tomatoes and tobacco lines transformed with a bacterial *LCYB* gene (*Erwinia herbicola*) remained unchanged (Wurbs *et al.*, 2007; Apel and Bock, 2009). In both tomato and tobacco transplastomic lines, higher LCYB activity was related to the higher tolerance to the herbicide CPTA. In order to reduce *LCYB* expression we used RNAi in order to obtain a wide range of transgene silencing and distinguish between harmful and non-harmful (for plant growth and development) reductions in the expression of the gene.

Here, by generating both transplastomic tobacco lines strongly expressing *DcLCYB1* and RNAi mutants repressing the tobacco LCYB enzyme to different residual levels, we will be able to conclude if *LCYB* expression is tightly related to growth regulation. Following our recently published results for nuclear *DcLCYB1* lines (Moreno *et al.*, 2020), we would expect that high *DcLCYB1* expression levels in our transplastomic lines could trigger a co-expression of key genes of carotenoid and carotenoid-related pathways, thus causing an increase in the content of key isoprenoids (e.g. β-carotene, xanthophylls, chlorophylls, GA, and ABA) and ultimately leading to higher growth and biomass. By contrast, reduced *NtLCYB* expression in our RNAi lines was hypothesized to trigger reduced expression of key genes of carotenoid and carotenoid-related pathways, thus causing a reduction in the content of key isoprenoids (e.g. β-carotene, xanthophylls, chlorophylls, GA, and ABA) and ultimately leading to reduced growth and biomass. If this is true, the reduced plant biomass might be due to these additional disturbances and impaired photosynthetic performance, which is directly related to the structural function of carotenoid in the photosynthetic apparatus. Hence, gene expression levels, pigment and hormone contents, biomass parameters, photosynthetic efficiency, chloroplast structure, and primary and secondary metabolism were evaluated in these lines. We show that plastid *DcLCYB1* expression leads to increased LCYB activity (indirectly measured as increased CPTA tolerance) and pigment content (β-carotene and violaxanthin), but not necessarily to increased biomass and photosynthesis, whereas reduced *NtLCYB* expression negatively affects plant physiology and development, primary and secondary metabolism, photosynthetic efficiency, and ultimately plant biomass.

## Materials and methods

### Plant material and growth conditions

Tobacco (*N. tabacum* cv. Petit Havana) wild type, RNAi, and transplastomic lines were raised from seeds germinated in Petri dishes containing Murashige and Skoog (MS) medium (Murashige and Skoog, 1962) supplemented with 30 g l^−1^ sucrose. Kanamycin (100 μg ml^−1^) was used for selection of nuclear-transgenic plants, and spectinomycin and streptomycin (500 μg ml^−1^) were used for selection of transplastomic plants. The leaf tissue for all the experiments (qPCR and pigment, metabolite, and hormone quantification) were taken from the fourth fully developed (bottom to the top) leaf from 6-week-old tobacco plants. For photosynthetic measurements, seedlings were transferred 14 d after germination to a soil–vermiculite mixture (2:1) and grown in a controlled-environment chamber at 350 μmol photons m^−2^ s^−1^ light intensity (16 h day, 22 °C, 75% relative humidity). For growth-related measurements (plant height, leaf surface, flower and leaf numbers), sampling, and seed production, plants were grown under greenhouse conditions.

### Transplastomic vector design and transformation

The complete coding sequence of the carrot *DcLCYB1* gene was synthesized by Thermo Fisher Scientific, and further amplified with primers designed to introduce 5′- and 3′-*Eco*RV restriction sites for incorporation into plastid expression vectors. The expression vector pDK325, containing a *Chlamydomonas reinhardtii* (*Cr*) *CrpsbA* promoter (*CrPpsbA*) coupled to the T7 phage *gene10 leader* (*T7g10L*) (Svab and Maliga, 1991) sequence upstream of the *Eco*RV site, and a *CratpA* terminator (*CrTatpA*), was used for creation of *Nt*-pJM36. The expression vector pDK326, containing the *CrPpsbA* and 5′-untranslated region and the *CrTatpA*, was used for creation of *Nt*-pJM37 vector. Both vectors included a chimeric *aadA* gene, including *aadA* and plastid expression elements (Svab and Maliga, 1993), for selection of transformed plants. Plant transformation was undertaken using the previously described biolistic protocols (Svab and Maliga, 1993; Ruf and Bock, 2011). Transformed plants were selected by growth on spectinomycin-containing medium (500 µg ml^−1^), and underwent several rounds of regeneration to obtain homoplasmic plants. Spontaneous spectinomycin-resistant lines were eliminated by double selection on medium containing both spectinomycin and streptomycin (500 µg ml^−1^ each) (Svab and Maliga, 1991; Bock, 2001). The homoplasmic state was confirmed by restriction fragment length polymorphism (RFLP) analysis. Briefly, total DNA was isolated by the CTAB method (Doyle and Doyle, 1990) from wild-type and transplastomic plants. *Bam*HI-digested samples were separated by agarose gel electrophoresis, transferred to Hybond XL membranes by capillary blotting, and hybridized using an α-[^32^P]dCTP-labelled probe targeting the *psaB* gene fragment (Fig. 1C). Homoplasmic transplastomic lines were rooted on hormone-free medium and subsequently transferred to the greenhouse for seed production.

### RNAi silencing vector design and transformation

Using the pENTR™ directional TOPO® Cloning Kit (Thermo Fisher Scientific), the PCR-derived *NtLCYB* fragment was cloned into pENTR/SD/D-TOPO® according to the manufacturer’s protocol. The PCR product (used for Gateway^TM^ cloning) was amplified with a forward primer containing CACC at its 5′ end (5′-*CACC*TTGTTGGATTGCCTCGACGCC-3′) to match the overhang in the cloning vector (GTGG; reverse primer 5′-CTCCACTTCTGCCAATATGCC-3′). This entry vector was then used to perform a Gateway recombination reaction generating the final expression vector pK7GWIWG2 (I) with the *NtLCYB* RNAi regions in sense and antisense orientation. The Gateway® LR cloning (Thermo Fisher Scientific) reaction was carried out following the manufacturer’s instructions. Transformation of the *NtLCYB* RNAi constructs into tobacco (*N. tabacum* cv. Petit Havana) was undertaken by *Agrobacterium tumefaciens*-mediated gene transfer using bacterial strain C58C1:pGV2260 (Rosahl *et al.*, 1987).

### Physiological measurements and plant biomass experiment

T_1_ generation homoplasmic *DcLCYB1* and T_1_ heterozygous *NtLCYB* RNAi seeds were used for physiological and biomass measurements as previously described (Moreno *et al.*, 2020). T_2_ generation homoplasmic *DcLCYB1* and T_3_ heterozygous *NtLCYB* RNAi seeds were used for germination (*n*=20), root length (*n*=6), and biomass quantification (*n*=6) experiments (10-day-old tobacco plants) in Petri dishes (repeated three times).

### RNA isolation, cDNA synthesis, and qPCR experiments

For transplastomic and RNAi lines, the fourth leaf from the bottom of the plants and the leaf with the most severe phenotype were sampled. Leaf tissue was frozen immediately in liquid nitrogen and ground to a fine powder. RNA extraction, cDNA synthesis, and qPCR experiments were performed as previously described (Moreno *et al.*, 2020), using the primers described therein. Three biological and three technical replicates per line were analysed. The relative transcript levels of each gene were determined using the formula (1+*E*)^−ΔΔ*C*t^where *E* is the binding efficiency of the primers (Pfaffl, 2001). All the primers used in this study were previously assessed and tested for expression analysis using at least two references genes (Schmidt and Delaney, 2010; Albus *et al.*, 2012; Kromdijk *et al.*, 2016; Moreno *et al.*, 2016; Armarego-Marriott *et al.*, 2019; Moreno *et al.*, 2020). In addition, expression analysis were performed following the same parameters and conditions as in Moreno *et al.* (2020), in which three different reference genes (*ACTIN*, *UBIQUITIN*, *PDF2*) were used. Therefore, in our current work only *Actin* transcript levels were measured as reference. Previously published primers that were used in this work can be found in Supplementary Table S1.

### UHPLC analysis of pigments

Plant pigments were extracted and subsequently analysed by Acquity UPLC™ H-Class System (Waters, Milford, MA, USA) equipped with an autosampler, quaternary solvent manager and a photodiode-array detection detector as described in Moreno *et al.* (2020). Five biological replicates per line were measured and data were analysed with Empower software (v. 3, Waters, Manchester, UK).

### ABA and GA measurements

Extraction and purification of ABA and GAs were performed using 15 mg (dry weight) leaf tissue following the procedure described in Turečková *et al*. (2009) and Urbanová *et al*. (2013). All data were processed using MassLynx™ software (v. 4.2, Waters, USA). Hormone levels were calculated on the basis of the standard isotope dilution method (Rittenberg and Foster, 1940).

### Hormone and inhibitor treatments

Hormone and inhibitor treatments were performed as previously described (Moreno *et al.*, 2020) but using liquid MS (Murashige and Skoog, 1962), supplemented with 1% sucrose, and 24-wells plates. Six tobacco seedlings per line were used in each treatment (*n*=6). Tobacco seedlings grown in agar MS medium (10-day-old) were transferred to liquid MS medium (with agitation) and treated for 7 d with hormones (GA_3_, GA_4_, ABA, 1 µM; GA_3_/ABA, 1 µM/0.66 µM; and GA_4_/ABA, 1 µM/0.66 µM) and inhibitors (paclobutrazol/PBZ, 1 µM). Six biological replicates for each transgenic line and wild type were grown in liquid MS medium in the 24-well plate to evaluate the effect of each hormone and hormone inhibitor treatment, and therefore each set of transgenic lines (transplastomic or RNAi lines) was compared separately.

### Extraction and phase separation for GC and LC analyses

Tobacco samples (fourth fully developed leaf from 6-week-old plants) were extracted using methyl tertiary butyl ether buffer as previously described (Salem *et al.*, 2016). After removal of the lipid phase, aliquots of 200 μl and 300 μl of the polar phase were transferred to new 1.5 ml Eppendorf tubes for GC-MS and LC-MS analysis, respectively. Samples were evaporated to dryness (speed-vac concentrator, Thermo Fisher Scientific) without heating.

### Analysis of primary metabolites from the methanol–water phase by GC-MS

Samples were derivatized and analysed as previously described (Lisec *et al.*, 2006; Caldana *et al.*, 2013). Mass chromatograms were processed and peak areas integrated using the software Xcalibur (v. 4.0, Thermo Fisher Scientific). Peak annotation was performed by matching the retention index relative to the fatty acid methylesters and mass spectra against an in-house reference library (Cuadros-Inostroza *et al.*, 2009). Results reported as log_2_ fold change were plotted as a heatmap using the pheatmap package (Kolde, 2019). The primary metabolite-reporting list following the recommendations described in Fernie *et al.* (2011) is provided in Supplementary Dataset S1.

### Analysis of secondary metabolites from the methanol–water phase by UPLC-MS

Samples were re-suspended in 200 μl methanol–water (1:1, v/v) and analysed as previously described (Giavalisco *et al.*, 2011). Data processing and statistical analysis was performed as previously described for primary metabolites. Peaks were annotated based on an accurate mass and elution profile of secondary metabolites, previously characterized in *Nicotiana attenuata* (Li *et al.*, 2016). The secondary metabolite-reporting list following the recommendations described by Fernie *et al.* (2011) is provided in Supplementary Dataset S2.

### Quantification of apocarotenoids using UHPLC-QQQ-MS/MS

Analysis of apocarotenoids from tobacco leaves was performed on a Vanquish™ Flex UHPLC System with an ACQUITY UPLC BEH C_18_ column (100×2.1 mm×1.7 mm) coupled with a QQQ-MS (TSQ Altis™ Triple Quadrupole Mass Spectrometer, Thermo Scientific) with a heated-electrospray ionization source according to the protocol modified from Mi *et al.* (2018). Briefly, tobacco leaves were harvested, lyophilized, and powdered. Approximate 25 mg tissue powder spiked with internal standards mixture (including D_3_-β-ionone, D_3_-β-apo-11-carotenal, D_3_-3-OH-β-apo-13-carotenone, D_3_-β-apo-13-carotenone, D_3_-β-apo-15-carotenal, D_3_-β-apo-14′-carotenal, D_3_-β-apo-12′-carotenal, D_3_-β-apo-10′-carotenal, and D_3_-β-apo-8′-carotenal; 2.5 ng of each standard) was extracted with methanol containing 0.1% butylated hydroxytoluene twice in an ultrasound bath, followed by centrifugation. The supernatant was collected and dried under vacuum. The residue was re-dissolved in 150 μl of acetonitrile–water (90:10, v/v) and filtered through a 0.22 µm filter for LC-MS analysis (Mi *et al.*, 2018). Apocarotenoid profiling was performed by using UHPLC-QQQ-MS/MS in selective reaction monitoring (SRM) mode. The SRM transition list is shown in Supplementary Dataset S3. Apocarotenoid standards (Buchem BV, Apeldoorn, Netherlands) including β-cyclocitral, 3-OH-β-ionone, β-ionone, 3-OH-β-apo-11-carotenal, β-apo-11-carotenal, 3-OH-β-apo-13-carotenone, β-apo-13-carotenone, 3-OH-β-apo-15-carotenal, β-apo-15-carotenal, β-apo-14′-carotenal, 3-OH-β-apo-12′-carotenal, β-apo-12′-carotenal, 3-OH-β-apo-10′-carotenal, β-apo-10′-carotenal, 3-OH-β-apo-8′-carotenal, and β-apo-8′-carotenal were used to validate the identification of apocarotenoids from tobacco leaves using UHPLC-MS.

### Photosynthesis measurements

Measurements of chlorophyll content and the Chl *a*/*b* ratio (fourth leaf from bottom to the top) were undertaken with a Jasco V-630 photometer (Jasco GmbH, Groß-Umstadt, Germany) in 80% (v/v) acetone (Porra *et al.*, 1989). Chl *a* fluorescence of intact plants was measured using a DUAL-PAM-100 instrument (Heinz Walz GmbH, Effeltrich, Germany) after 30 min of dark adaptation. Light intensity was increased stepwise from 0 to 2500 µE m^−2^ s^−1^, with a measuring time of 150 s per step under light-limited conditions and of 60 s under light-saturated conditions. Light-response curves of non-photochemical quenching (qN; Krause and Weis, 1991), the redox state of the PSII acceptor side (qL; Kramer *et al.*, 2004), and of the donor-side limitation of PSI (Y(ND); Schreiber and Klughammer, 2016) were determined. Linear electron transport was corrected for leaf absorptance, which was calculated from leaf transmittance and reflectance spectra as 100% minus transmittance (%) minus reflectance (%). Spectra were measured between 400 and 700 nm wavelengths using an integrating sphere attached to a photometer (V650, Jasco Inc.). The spectral bandwidth was set to 1 nm, and the scanning speed was 200 nm min^−1^.

### Microscopy techniques

Leaf samples at similar developmental stages were fixed for a period of 1 h in a solution containing 45% ethanol, 5% glacial acetic acid, and 5% formaldehyde. The samples were subjected to ethanol dehydration series for observing leaf anatomy. Subsequently the samples were infiltrated with 1% Hardener I in Technovit 7100 and incubated for up to 24 h after a short 2 h infiltration with Technovit 7100. The embedding was carried out using 15 parts of infiltration solution with 1 part of Hardener II in molds and polymerized at room temperature overnight. Sectioning was undertaken using a rotary microtome (Leica RM2265) and sections of 5 µm were prepared and stained with 0.05% toluidine blue and visualized and recorded using an Olympus light microscope (BX51).

For chloroplasts counting, leaves were selected and sampled using the same procedure as for the leaf cross sections (see above). To each leaf piece 1.5 ml fixation solution (TissuePrep Buffered 10% Formalin; Electron Microscopy Sciences; Hatfield, PA, USA) was added and incubated for 1.5 h under vacuum. Subsequently, the fixation solution was replaced by fresh solution and the tubes were constantly inverted for 2 h. Then, samples were incubated at 4 °C overnight and afterwards heated for 3 h at 60 °C. Images of fixed leaf cells were taken using an Olympus light microscope (BX51). Chloroplasts of 20 cells per line were counted.

For chloroplast ultrastructural analysis, leaf tissue (of the fourth leaf of the transplastomic and the most variegated of the RNAi lines) was fixed in 2.5% glutaraldehyde supplemented with 0.2 M sodium cacodylate for a period of 8 h. The samples were then incubated with 2% osmium tetroxide for a period of 4 h. The samples were rinsed and taken through dehydration steps and embedded in Epon resin using standard protocols (Austin and Staehelin, 2011). Sections of 1–2 µm were cut by diamond knife, and stained (2% uranyl acetate and lead citrate) prior to imaging using a Zeiss EM 912 Omega transmission electron microscope.

### Statistical analysis

Three main experiments with transgenic plants (transplastomic and RNAi lines) growing under greenhouse or fully controlled conditions (phytochamber) were performed in this work. Transgenic lines were compared in two groups (transplastomic and RNAi lines) due to the high number of lines and biological replicates. Thus, transplastomic and RNAi lines were always grown and compared in two different groups with their own wild-type control. The same leaf tissue (fourth leaf bottom to top) from 6-week-old tobacco was used to perform all the molecular analysis. The first experiment included a set of tobacco plants used for molecular analysis such as qPCR (*n*=3 and three technical replicates), pigment quantification via UPLC (*n*=5), and hormone (*n*=5 and three technical replicates) quantification. In addition, for primary and secondary metabolites (*n*=6) statistical analysis was performed in R (R Core Team, 2018) using an unpaired Wilcoxon test with the default parameters within the function compare_means of ggpubr package (Kassambara, 2018). The second main experiment consisted of tobacco plants used for physiological parameters and biomass quantification (*n*=5) of tobacco plants grown in the greenhouse. Due to the high number of transgenic lines and biological replicates, the experiments were performed with 3 d of difference, starting with the transplastomic lines and then the RNAi lines (each transgenic group with their own wild type). The third main experiment consisted of a set of tobacco plants used for photosynthetic analysis (*n*=3–8). Plants were grown in a controlled-environment chamber in a consecutive manner due to space constrains (first transplastomic and then RNAi lines, each group with its own wild-type control). In a fourth experiment, tobacco seedlings grown in solid MS medium were used. Germination rate (*n*=20), root length (*n*=6), and biomass (*n*=6) experiments were performed in 10-day-old tobacco seedlings. These experiments were repeated three times in different Petri dishes. For the last (fifth) experiment, corresponding to the hormone and hormone inhibitors experiments, 10-day-old tobacco seedlings growing on half-strength solid MS medium were transferred to a 24-well plate. Each row consisted of six wild type, and six replicates of the three transplastomic or RNAi lines, and therefore the plants within a plate were always compared with each other (these plants came from the same Petri dish where they grew for 10 d). Due to the experimental design for the five different data sets of plants, an unpaired, two-tailed Student’s *t*-test was performed to compare each set of transgenic lines with the wild type (unless otherwise stated). GraphPad Prism 5.0 software was used to prepare the figures and perform the *t*-tests.

## Results

### Generation of homoplasmic *Daucus carota* lycopene-β-cyclase1 expressing lines

The full-length carrot *DcLCYB1* gene was inserted into the pDK325 and pDK326 vectors to generate the pJM36 and pJM37 chloroplast transformation vectors, respectively (Fig. 1B). The pJM36 and pJM37 vectors contained the *Chlamydomonas reinhardtii* (*Cr*) *PsbA* promoter (*CrPpsbA*) and *CrAtpA* terminator (*CrTatpA*), flanking the synthetic *DcLCYB1* gene, and a chimeric spectinomycin resistance gene (*aadA*, an aminoglycoside adenylyltransferase) as selectable marker. In addition, the *CrPpsbA* in the pJM36 vector was fused to the strong translation initiation signals derived from the *gene10* leader of coliphage T7 (*T7g10L*) (Kuroda and Maliga, 2001). Both constructs were introduced into the genome of *Nicotiana tabacum* cv. Petit Havana by biolistic chloroplast transformation (Svab and Maliga, 1993; Ruf *et al.*, 2001; Ruf and Bock, 2011), and transplastomic plants were selected on spectinomycin-containing medium (see Materials and methods). Resistance to spectinomycin can arise from expression of the *aadA* marker gene, but also from spontaneous mutations proximal to the tRNA binding region of the 16S rRNA. As these spontaneous mutants are still sensitive to streptomycin, which, however, is detoxified by *aadA* (Svab and Maliga, 1991), mutant lines isolated on spectinomycin were further tested for streptomycin resistance.

Interestingly, only two *aadA*-containing positive (spectinomycin and streptomycin resistant plants) and 20 spontaneous mutant (plants resistant to spectinomycin but sensitive to streptomycin) (Svab and Maliga, 1991) lines were obtained with the pJM36 vector (*T7g10L*; Supplementary Table S2). By contrast, 23 positive transformants and only 18 spontaneous mutants were obtained with the pJM37/*LCYB1* vector (Supplementary Table S2). The high number of spontaneous mutants relative to true mutants (Supplementary Table S2) suggests the possibility of a toxic effect attributed to the transformation with the pJM36/*LCYB1* vector (Svab and Maliga, 1991). Moreover, one of the positive pJM36/*LCYB1* lines died during the tissue culture process, supporting the idea of a toxic effect caused by the extremely high expression of the transgene. Due to this, we performed further analysis with only one pJM36/*LCYB1* line. From the obtained lines, seven putative transformants (one transformed with pJM36 and six with pJM37) were purified to homoplasmy by passing them through additional regeneration cycles under antibiotic selection. To confirm chloroplast transformation, proper integration of the transgene via homologous recombination and homoplasmy of the transplastomic lines, RFLP analysis using Southern blotting was performed (Fig. 1C). *Bam*HI digestion of the wild type DNA generated a single band of 4.5 kb (Fig. 1C), while for the seven transgenic lines a band of 7.9 kb (consistent with expected length following the insertion of the transgene and the respective plastid expression elements), but no 4.5 kb wild type band, was obtained (Fig. 1C). In addition, and as an ultimate support for the homoplasmic state, a large-scale inheritance test was conducted, involving germination of T_1_ seeds on spectinomycin-containing medium (Fig. 1D) (Bock, 2001). No appearance of antibiotic-sensitive seedlings, as could be observed for the wild type, was observed in any of the analysed lines (Fig. 1D), suggesting a homoplasmic state in the *DcLCYB1* transplastomic lines. Homoplasmic T_1_*Nt*-pJM37/*LCYB1* plants were indistinguishable from the wild-type control at the seedling stage, indicating that integration and expression of the transgene was phenotypically neutral at early stages of development. By contrast, it seems that very high *LCYB1* expression in the *Nt*-pJM36/*LCYB1* plants caused a toxic effect (probably causing an imbalance in phytohormone content, see Discussion) that impaired plant development impeding the generation of more transplastomic lines.

### Increased *DcLCYB1* expression results in increased pigment content in transplastomic *DcLCYB1* lines

**Fig. 2.**
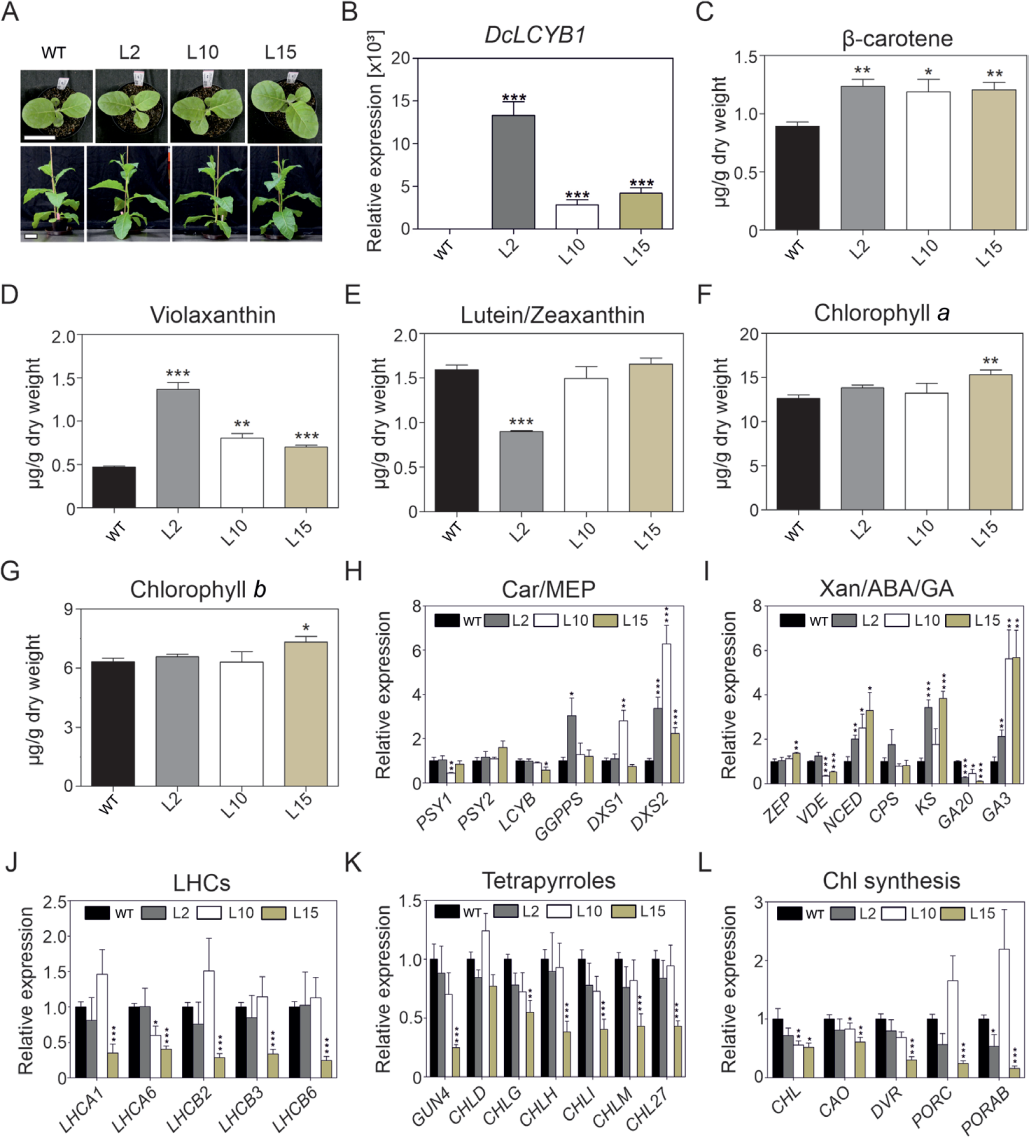
Highly increased *DcLCYB1* expression causes no changes in phenotype but increases pigment content and affects gene expression in tobacco plants. (A) Two-week-old (upper) and 4-week-old (lower) transplastomic and wild-type plants. Scale bar: 10 cm. (B) *DcLCYB1* expression levels measured by qPCR. (C–G) Pigment content (carotenoids and chlorophylls) measured by UPLC. (H–L) Gene expression analysis of carotenoid and carotenoid-related pathways. The expression of *Actin* as a stable reference gene was used for normalization. Increases, decreases and/or no change in all three lines for the majority of the genes involved in carotenoid, MEP, chlorophyll, GA, LHC, and tetrapyrrole pathways were observed. Columns and bars represent the means and SEM for the qPCR (three biological replicates and three technical replicates) and UPLC (five biological replicates) experiments. Unpaired two-tailed Student’s *t*-test was performed to compare transgenic lines with the wild type. *P<0.05, **P<0.001, ***P<0.0001. ABA, abscisic acid; Car, carotenoids; Chl, chlorophyll; GA, gibberellins; L2, pJM36-2; L10, pJM37-10; L15, pJM37-15; LHC, light harvesting complex; MEP, 2-*C*-methyl-D-erythritol 4-phosphate; WT, wild type; Xan, xanthophylls.

From the seven confirmed homoplasmic lines, we choose the lines L2 (pJM36), L10, and L15 (pJM37) to perform further molecular analysis. All three lines showed a phenotype similar to the wild type after 2 and 4 weeks of growth under greenhouse conditions (Fig. 2A). Line L2 was transformed with the pJM36 vector containing the expression element *T7g10L* (Fig. 1A), and therefore *DcLCYB1* expression was much higher (~12 000-fold relative to the wild type) than that of line L10 and L15, which were transformed with the pJM37 vector without the *T7g10L* expression element (~5000-fold higher relative to the wild type; Fig. 2B). Despite the differences in expression level, all three lines had an increase of approximately 30% in β-carotene content (Fig. 2C). This correlated with the increased tolerance to CPTA, which specifically inhibits LCYB activity (Schuetz and Baldwin, 1958; Tao *et al.*, 2004), observed in the transplastomic lines subjected to CPTA treatment (Supplementary Fig. S1). Wild-type plants showed chlorosis/leaf variegation and stunted growth, while lines L10 and L15 only showed moderate variegation and line L2 remained completely green after 8 d of CPTA treatment (Supplementary Fig. S1C). Enhanced CPTA tolerance indirectly suggests increased LCYB activity in the transgenic lines. In addition, violaxanthin content was increased in all lines, about 190%, 82%, and 48% for line L2, L10, and L15, respectively (Fig. 2D). Additionally, line L2, which has the highest *DcLCYB1* expression, showed a decrease to 56% of the wild type in lutein/zeaxanthin content (Fig. 2E). Moreover, chlorophyll *a* and *b* contents were significantly increased in line L15 (21% and 16%, respectively; Fig. 2F, G).

Recently, it was shown that expression of *DcLCYB1* in tobacco (cv. Xanthi) leads to a general increase in transcripts of carotenoid and carotenoid-related genes (Moreno *et al.*, 2020). However, such a general positive impact on carotenoid and carotenoid-related pathways was not observed in the transplastomic lines (Fig. 2H–L; Supplementary Fig. S2A, B). Transcript accumulation of the majority of the genes analysed did not increase (it was unchanged or reduced), possibly due to a negative impact of extremely high *DcLCYB1* expression (feedback regulation) on another isoprenoid pathway. However, some changes were consistently significant in all lines: steady state accumulation of deoxyxylulose 5-phosphate synthase2 (*DXS2*) transcripts was highly increased, 3.4-, 6.3-, and 2.4-fold in lines L2, L10, and L15, respectively (Fig. 2H). Additionally, 9-*cis*-epoxycarotenoid dioxygenase (*NCED*) transcript accumulation was increased, 2-, 2.5-, and 3.3-fold, respectively, in the three lines (Fig. 2I). Transcript accumulation of genes encoding key enzymes involved in GA biosynthesis, gibberellin 20- and 3-oxidases (*GA20ox* and *GA3ox*), was reduced (3.6-, 2.1-, and 9.1-fold in L2, L10, L15, respectively) and increased (2.1-, 5.6-, and 5.7-fold in L2, L10, L15, respectively) in all the lines, respectively (Fig. 2I). Furthermore, the majority of transcripts of tetrapyrrole and chlorophyll biosynthesis genes and genes encoding LHC subunits were decreased in line L15 (Fig. 2J–L). Transcript abundance of genes encoding subunits of PSI and PSII and the cytochrome *b*_*6*_*f* (cyt *b*_*6*_*f*) complex were not consistently changed across all lines (Supplementary Fig. S2A, B).

### Reduction in *NtLCYB* expression leads to a decrease in pigment content and negatively impacts gene expression in carotenoid and carotenoid-related pathways

**Fig. 3.**
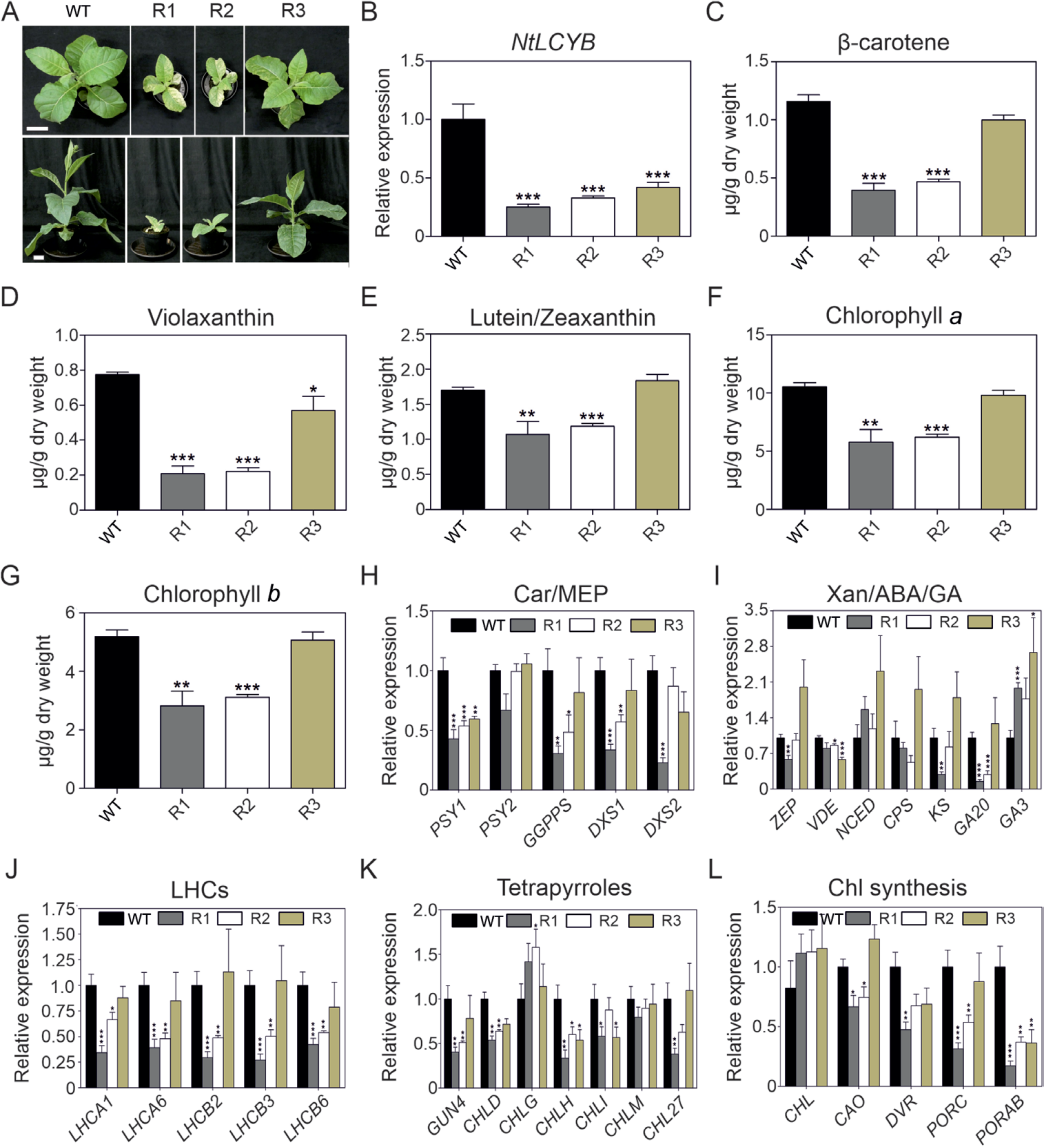
Reduced *NtLCYB* expression causes decreases in plant growth, pigment content, and gene expression in tobacco plants. (A) Side and top view from 4-week-old RNAi and wild type plants. Scale bar: 10 cm. (B) *NtLCYB* expression levels measured by qPCR. (C–G) Pigment content (carotenoids and chlorophylls) measured by UPLC. (H–L) Gene expression analysis of carotenoid and carotenoid-related pathways measured by qPCR. The expression of *Actin* as a stable reference gene was used for normalization. A general decrease in transcript abundance of all three lines for the majority of the genes involved in carotenoid, MEP, chlorophyll, and GA pathways was observed. Decrease in transcript accumulation of genes encoding LHC subunits and enzymes involved in tetrapyrrole biosynthesis was observed. Columns and bars represent the means and SEM for the qPCR (three biological replicates and three technical replicates) and UPLC (five biological replicates). Unpaired two-tailed Student’s *t*-test was performed to compare transgenic lines with the wild type. Letters represent statistical differences among the lines. *P<0.05, **P<0.001, ***P<0.0001. ABA, abscisic acid; Car, carotenoids; Chl, chlorophyll; GA, gibberellins; LHCs, light harvesting complex; MEP, 2-*C*-methyl-D-erythritol 4-phosphate; WT, wild type; Xan, xanthophylls.

Reduction in *NtLCYB* gene expression was accomplished through silencing using RNAi. Reduced *NtLCYB* expression levels resulted in a variegated phenotype for approximately half of the transformants (10) growing in sugar-supplemented medium (Supplementary Fig. S3A). These lines were able to later grow photoautotrophically in soil under greenhouse conditions (Supplementary Fig. S3B). The other half presented a white/pale green phenotype when grown in synthetic medium and were not able to survive after transfer to soil (Fig. S3A, C). These phenotypes suggest the importance of proper β-carotene levels for plant viability. The T_1_ generation of *NtLCYB* RNAi lines was obtained and three independent transgenic lines with different strengths of variegation (R1>R2>R3) were chosen to perform further molecular analysis. Lines R1, R2, and R3, showing 25%, 33%, and 42% residual expression of *NtLCYB* gene, showed delayed development compared with the wild type (Fig. 3A, B). The relative fitness and residual *NtLCYB* expression level are reflected in the reduction of β-carotene content, which decreased ~65% for the line with the strongest phenotype (R1) and ~15% for the line with the weakest phenotype (R3; Fig. 3C). Furthermore, violaxanthin content was significantly reduced in all lines (Fig. 3D). In addition, lutein/zeaxanthin and chlorophyll *a* and *b* content were decreased in R1 and R2 but remain comparable to those of the wild type in R3 (Fig. 3E–G).

In order to understand the reductions in carotenoid and chlorophyll content, we performed gene expression analysis to measure transcript abundance of genes encoding enzymes involved in carotenoid and chlorophyll biosynthesis. Carotenoid, chlorophyll, and GA pathways share a common biosynthetic precursor, geranyl geranyl diphosphate (GGPP), which is produced via the MEP pathway. For this reason, key genes of the MEP and GA pathways were also analysed. Expression of genes with products involved in the production of GGPP such as *GGPP synthase* (*GGPPS*), *DXS1*, and *DXS2* were strongly reduced in R1 and R2 (Fig. 3H). Moreover, *PSY1* transcript was reduced ~50% for all lines while *PSY2* transcript remain unchanged.

Transcript abundance of other carotenoid biosynthetic genes such as *ZEP* and *VDE*, which encode enzymes involved in the xanthophyll cycle, were not consistently reduced in the RNAi lines (*ZEP* was reduced in R1 while *VDE* was reduced in R2 and R3; Fig. 3I). In addition, transcript of *NCED*, which encodes the enzyme catalysing the first step of abscisic acid biosynthesis, was unchanged in the RNAi lines (Fig. 3I). *CPS* and *KS* (with products involved in the early stage of the GA pathway) transcripts remain mostly constant in all the lines (significant decrease of *KS* in R1; Fig. 3I). Interestingly, *GA20ox* expression was decreased in R1 and R2 (Fig. 3I). It was previously reported that the expression of *GA20ox* has a strong impact on plant height, with higher expression leading to larger plants and lower expression resulting in dwarf phenotypes (Davière and Achard, 2013). Therefore, these changes may partially account for the phenotypes observed in line R1 and R2 compared with the wild type (Coles *et al.*, 1999; Vidal *et al.*, 2001). Moreover, GA3-OXIDASE is encoded by *GA3ox* acting downstream of the *GA20ox* converting inactive GA into bioactive GA such as GA_1_ and GA_4_ (Gallego-Giraldo *et al.*, 2008; Hedden and Thomas, 2012). In our RNAi lines, *GA3ox* was increased in all transgenic lines (Fig. 3I), perhaps as a response to counteract the GA deficit.

The accumulation of transcripts encoding five LHCs from PSI and PSII (*LHCA1*, *LHCA6*, *LHCB2*, *LHCB3*, and *LHCB6*) was reduced in the two strongest RNAi lines while in R3 LHC transcript accumulation was not changed (Fig. 3J). In addition, the transcript accumulation of genes encoding enzymes involved in tetrapyrrole biosynthesis was decreased in R1 (*GUN4*, *CHLD*, *CHLH*, *CHLI*, *CHL27*), R2 (*GUN4*, *CHLD*, *CHLH*), and R3 (*CHLH*, *CHLI*; Fig. 3K). Moreover, accumulation of the transcripts of genes encoding enzymes involved in chlorophyll biosynthesis such as *CAO* (R1 and R2), *DVR* (R1), *PORC* (R1 and R2), and *PORAB* (R1, R2 and R3) was reduced (Fig. 3K). Strikingly, the majority of transcripts encoding subunits of PSI, PSII, and cyt *b*_*6*_*f* were decreased in R1 (19 out of 20), R2 (13 out of 20), and R3 (seven genes) lines (Supplementary Fig. S2C, D). These results indicate a relationship between the reduction of the *NtLCYB* transcript, the strength of the phenotype, and the reduction in the different key photosynthesis and photosynthesis-related genes.

### Altered *LCYB* expression influences plant biomass in tobacco plants

As mentioned earlier, transplastomic lines expressing the *DcLCYB1* gene were phenotypically indistinguishable from the wild type (Fig. 2A), while 4-week-old *LCYB* RNAi lines showed leaf variegation, delayed development, and reduced growth (Fig. 3A). Moreover, at the end of the tobacco life cycle transplastomic lines were similar in height to the wild type (Supplementary Fig. S4A). By contrast, at the end of the plant life cycle (3-month-old plants), R2 and R3 plants had a comparable height to wild type plants while R1 remained dwarfed and produced fewer flowers (Supplementary Fig. S4B; Supplementary Table S3).

**Fig. 4.**
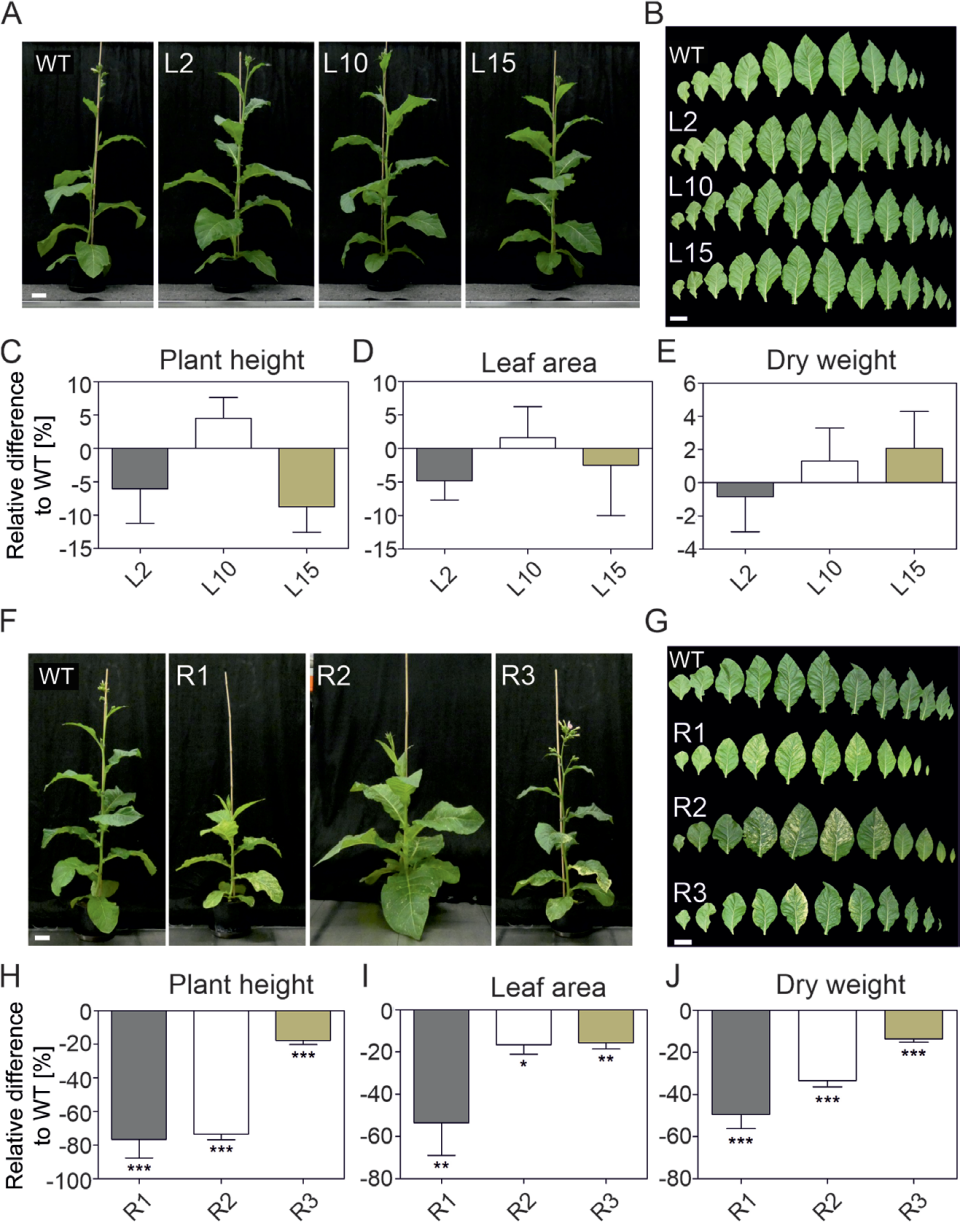
Plant productivity of transplastomic *DcLCYB1* and *NtLCYB* RNAi lines. (A) Side view of 5-week-old wild type and transplastomic tobacco plants. (B) Leaf series of 5-week-old wild type and transplastomic lines. (C–E) Plant height, leaf area, and dry weight biomass of 4-week-old transplastomic plants. (F) Side view of 5-week-old wild type and RNAi tobacco plants. (G) Leaf series of 5-week-old wild type and RNAi lines. (H–J) Plant height, leaf area, and dry weight biomass of 4-week-old RNAi plants. Columns and bars represent the means and SEM of five biological replicates. Unpaired two-tailed Student’s *t*-test was performed to compare transgenic lines with the wild type. *P<0.05, **P<0.001, ***P<0.0001. Scale bar: 10 cm. WT, wild type.

In order to analyse the relationship between *LCYB* transcript/β-carotene production and plant biomass, we performed physiological and biomass measurements with transplastomic *DcLCYB1* and *NtLCYB* RNAi lines containing increased and reduced *LCYB* transcript levels (Fig. 4). Transplastomic plants visibly resembled the wild type (Fig. 4A, B) and showed no differences in plant height or leaf area through different time points of their development (Fig. 4C–E; Supplementary Fig. S5A–D). By contrast, the RNAi lines showed a variegated-plant phenotype and delayed development (Fig. 4F), most pronounced in R1, then R2 and R3 (Fig. 4G–J). Interestingly, the variegated phenotype not only appeared in the leaves but also in stem and capsules of the two strongly affected RNAi lines (R1 and R2; Supplementary Fig. S4C, D). Physiological parameters such as plant height, leaf area, and plant biomass were drastically reduced in all three RNAi lines, with more pronounced reductions in the mutants with the stronger reductions, R1 and R2 (Fig. 4H–J). Furthermore, quantification of plant height, leaf area, leaf number, and internodal space through development supports our observation of the different phenotypic strength of each line (Supplementary Fig. S5E–H). This phenotype was also observed in the T_3_ generation of tobacco lines, in which R1 was even more affected than the parental R1 plants, probably due to homozygosity in this line (Supplementary Fig. S6A–C).

### Altered *LCYB* expression influence hormone metabolism and apocarotenoid synthesis in transgenic tobacco plants

**Fig. 5.**
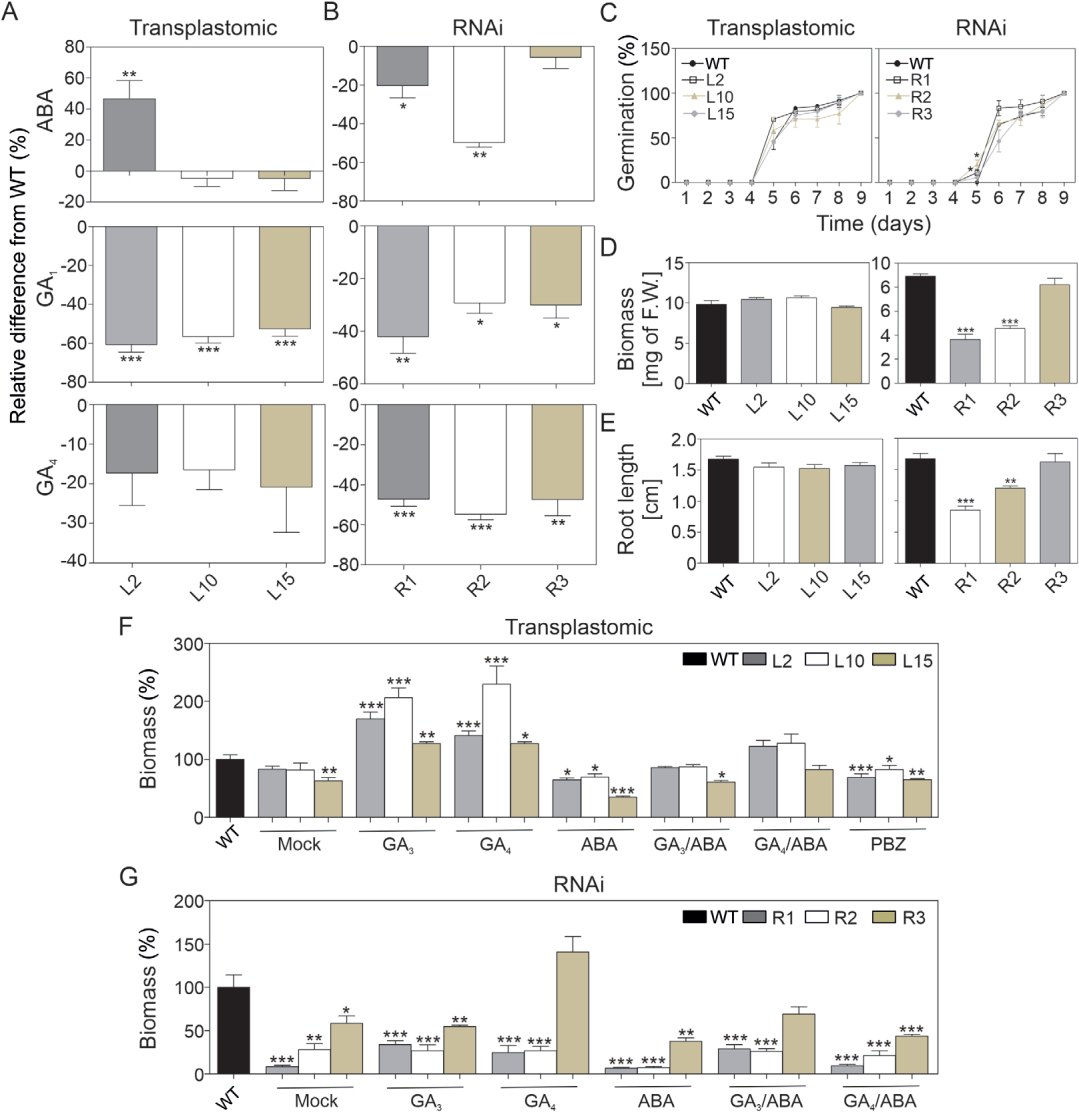
Hormone metabolism and its effect on plant physiology. (A) Abscisic acid and gibberellin content in transplastomic lines. (B) Abscisic acid and gibberellin (GA_1_ and GA_4_) content in RNAi lines. Columns and bars represent the means and SEM of five biological replicates and three technical replicates. (C) Seed germination in *DcLCYB1* transplastomic and *NtLCYB* RNAi lines (three independent plates, *n*=20). (D) Biomass of 10-day-old *DcLCYB1* transplastomic and *NtLCYB* RNAi lines (three independent plates, *n*=6). (E) Root length of 10-day-old *DcLCYB1* transplastomic and *NtLCYB* RNAi lines (three independent plates, *n*=6). (F) Hormone and inhibitor treatments in 10-day-old *DcLCYB1* transplastomic lines. (G) Hormone and inhibitor treatments in 10-day-old *NtLCYB* RNAi lines. Tobacco seedlings grown in agar MS medium (10-day-old) were transferred to liquid MS medium (with agitation) and treated for 7 d with hormones (GA_3_, GA_4_, ABA, 1 µM; GA_3_/ABA, 1 µM/0.66 µM; GA_4_/ABA, 1 µM/0.66 µM) and inhibitors (paclobutrazol/PBZ, 1 µM). Biomass was calculated as percentage for each treatment normalized to wild type as 100% in each respective treatment (*n*=6 for each transgenic line and wild type). Unpaired two-tailed Student’s *t*-test was performed to compare transgenic lines with the wild type (*P<0.05, **P<0.001, ***P<0.0001). ABA, abscisic acid, GA, gibberellins, GA_3_, gibberellin A_3_; GA_4_, gibberellin A_4_; PBZ, paclobutrazol; WT, wild type.

Hormone content was altered in both transplastomic and RNAi lines. Interestingly, ABA content was increased only in L2, the line with the highest *DcLCYB1* expression levels (Fig. 5A). GA1 was reduced ~60% in all three lines while GA4 remain unchanged (Fig. 5A). ABA (R1 and R2, ~20–40%), GA_1_ (~30%), and GA_4_ (~40–55%) contents were strongly reduced in the RNAi lines (Fig. 5B). Due to the changes observed in GA and ABA content in transplastomic *DcLCYB1* and *LCYB* RNAi lines, we measured ABA and GA metabolic intermediaries to gain further insights into GA and ABA metabolism. As expected, various GA and ABA metabolic intermediaries were affected in transplastomic (Supplementary Fig. S7) and RNAi lines (Supplementary Fig. S8). These results suggest that *LCYB* expression can modify hormone content, and therefore influence plant development and physiology. For instance, unchanged or slightly increased ABA levels in the transplastomic lines did not influence seed germination or plant biomass at the seedling stage (Fig. 5C–E) as was previously reported for increased ABA content (Garciarrubio *et al.*, 1997). By contrast, reduced ABA levels in R1 and R2 were reflected in early seed germination, reduced plant biomass, and shorter primary root length in tobacco seedlings compared with the wild type (Fig. 5C–E). To further dissect the hormonal effect on our plants we designed an experiment in which 10-day-old tobacco seedlings grown on agar MS medium were transferred to liquid MS medium in 24-well plates, and were subjected to hormone and inhibitor treatments for 7 d. Interestingly, results from hormone and inhibitor treatments shed light on how these tobacco lines can achieve higher/lower biomass (Fig. 5F, G). Transplastomic L2 and L10 lines treated with water (mock) showed the same plant biomass and architecture as the wild type, while the biomass of L15 was slightly lower (Fig. 5F; Supplementary Fig. S9A). GA_3_ and GA_4_ treatments resulted in increased biomass in all lines and the wild type, while ABA treatment resulted in decreased biomass. Under treatment with paclobutrazol, a specific inhibitor of GA synthesis, biomass of the transplastomic lines was also reduced, relative to the wild type (Fig. 5F). In addition, combinations of GA_3_ and GA_4_ with ABA did not alter plant biomass in the transplastomic lines. Water-treated (mock) RNAi lines showed reduced biomass compared with the wild type and confirmed the observed phenotype strength observed under greenhouse conditions (Figs 5G, 3A; Supplementary Fig. S9B). Interestingly, in the most affected RNAi lines, R1 and R2, neither GA_3_ or GA_4_ nor combinations of GA_3_ and GA_4_ with ABA treatments could rescue the phenotype to the wild-type level (Fig. 5G). In addition, RNAi lines showed reduced biomass after ABA treatment. Taken together these results suggest a slightly and profoundly altered hormonal balance in the transplastomic and RNAi lines, respectively.

β-Carotene-derived apocarotenoids (e.g. β-cyclocitral and zaxinone) play roles as growth (promoting) regulators in Arabidopsis, rice, and tomato (Dickinson *et al.*, 2019; Wang *et al.*, 2019); because carotenoid levels were altered in the *DcLCYB1* and *LCYB* RNAi lines (Figs 2C–E, 3C–E), we profiled apocarotenoid accumulation to determine if some of these molecules were also altered and thus may contribute to the growth phenotype observed in our tobacco lines. Due to the similar changes in carotenoid content obtained in transplastomic lines, we chose only one transplastomic line (L15) (Fig. 2C–E) for profiling, as well as R1, R2, and R3 RNAi lines because they showed different reductions in carotenoid content (Fig. 3C–E). Using a UHPLC-MS method (Mi *et al.*, 2018) we quantified different non-hydroxylated and hydroxylated apocarotenoids (Supplementary Fig. S10A, B). Despite increases in β-carotene and violaxanthin in the transplastomic lines, apocarotenoid levels remain the same as those in the wild type (Supplementary Fig. S10A, B). From the RNAi lines, only R1 (the most affected line at the phenotypic and molecular level) showed significant changes in apocarotenoid content including increases in β-apo-8′-, β-apo-10′-, β-apo-12′-, and 3-OH-β-apo-8′-carotenal. In addition, other apocarotenoids, such as β-apo-11-, β-apo-15-, 3-OH-β-apo-10′-, 3-OH-β-apo-11-, 3-OH-β-apo-14′-, and 3-OH-β-apo-15-carotenal, showed slight but significant reductions (Supplementary Fig. S10A, B). Although β-cyclocitral (β-cc) did not show a significant reduction, 3-OH-β-apo-13-carotenone (zaxinone), another growth (promoting) regulator in rice (Wang *et al.*, 2019), was strongly reduced in the most affected line (~30%, *P*<0.005). These results suggest that (i) strong reductions in carotenoid accumulation cause strong changes in apocarotenoid content, and (ii) zaxinone might be contributing to the observed growth phenotype in the RNAi lines.

### Chloroplast structure and photosynthetic analysis in *DcLCYB1* transplastomic and *NtLCYB* RNAi lines

In order to understand how phenotypic changes were present at a microscopic level (e.g. leaf structure), we performed light microscopy on sectioned leaf tissue of transplastomic and RNAi lines (Supplementary Fig. S11). We selected two transplastomic lines, one with the strongest *LCYB1* expression (L2) and another with moderate expression (L15), and in the case of the RNAi lines in terms of phenotype (yellow tissue/green tissue). As R1 and R3 exhibit a strong and moderate variegated-leaf phenotype, respectively, we decided to examine both green and yellow tissue independently.

**Fig. 6.**
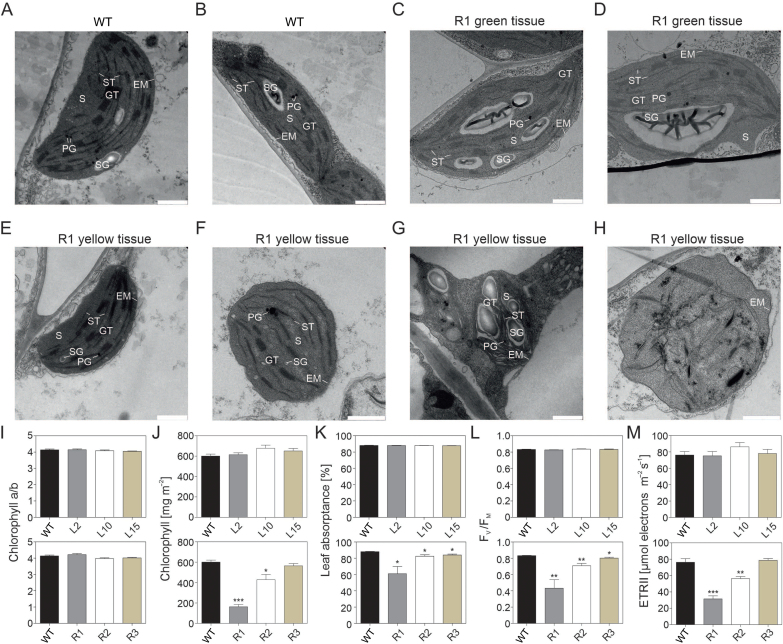
Chloroplast ultrastructure and photosynthetic analysis of *DcLCYB1* transplastomic and *NtLCYB* RNAi lines. Transmission electron microscopy images of chloroplasts of wild type plants and green and yellow sectors of 4-week-old R1 lines. (A, B) Wild type chloroplasts. (C, D) Chloroplasts in the green tissue of R1 leaves are similar to those of the wild type, but differences in starch granule number, apparent size, and shape can be observed. (E–H) A wide range of phenotypes were observed in the yellow sectors of R1 leaves (wild type-like, 23.3%; round, altered chloroplast morphology with impaired thylakoid structure, 5.6%; and unstructured and round chloroplasts 71.1%). (I–M) Photosynthetic parameters in transplastomic (upper panel) and RNAi (bottom panel) lines: (I) Chlorophyll *a*/*b* ratio; (J) chlorophyll content; (K) leaf absorptance; (L) maximum quantum efficiency of PSII in the dark-adapted state (*F*_v_/*F*_m_); and (M) ETRII, linear electron flux capacity, which was corrected for leaf absorptance. Transplastomic and RNAi lines were 4 weeks old. Columns and bars represent the means and SEM of three to eight biological replicates. Unpaired two-tailed Student’s *t*-test was performed to compare transgenic lines with the wild type. *P<0.05, **P<0.001, ***P<0.0001. EM, envelope membranes; GT, grana thylakoids; PG, plastoglobules; S, stroma; SG, starch granule; ST, stroma thylakoids; WT, wild type. Scale bar: 1000 nm.

In the transplastomic lines and in the RNAi line R3, no changes in the arrangement of cell layers in leaf cross sections were observed compared with the wild type (Supplementary Fig. S11A–C, F, G). By contrast, in yellow tissue of the strong variegated R1 line, palisade cells were smaller with reduced spacing between the cells (Supplementary Fig. S11E). Furthermore, a significantly increased number of chloroplasts was observed in the transplastomic lines (*P*<0.05) and decreased number in the RNAi lines (*P*<0.0001; Supplementary Table S4). To gain further insight into chloroplast ultrastructure, we performed transmission electron microscopy of wild type, R1 (strong phenotype), and R3 (mild phenotype) leaves. We observed that wild-type chloroplasts were organized side by side against the cell contour or densely packed in some regions of the cells. The green sectors of R1 leaves did not have a significantly different number of chloroplasts (16 per 1000 μm^2^) from the wild type (19 per 1000 μm^2^) and they had similarly positioned chloroplasts (Supplementary Fig. S12A–D; Supplementary Table S4). Chloroplast structure and organization was similar in R1 and R3, but the mild R3 line showed alleviated effects compared with the strongest R1 line. Green sectors of R1 and R3 leaves exhibited wild-type-like chloroplasts (Fig. 6C, D; Supplementary Fig. S12A–D, G–J). Yellow sectors of the R1 and R3 leaves showed four different types of chloroplast shapes: wild-type-like (30% and 52%, respectively), round (41% and 39%, respectively; chloroplasts not present close to the cell contour unlike the wild type), chloroplasts with several starch granules and an impaired thylakoid structure (16% and 6%, respectively), and chloroplasts with completely altered structure (14% and 4%, respectively; Fig. 6E–H; Supplementary Fig. S12E, F, K–N). By contrast, yellow sectors of R1 leaves had only a very few chloroplasts, which were either not present close to the cell contour, unlike the wild type, or unstructured (Supplementary Fig. S12E, F).

In order to better understand the impact of altered leaf and chloroplast structure on plant growth, we measured several photosynthetic parameters. Measurements were always performed on the youngest fully expanded leaves, to avoid effects of leaf shading or from the onset of leaf senescence. In transplastomic plants in their vegetative state (before flower production), the chlorophyll *a*/*b* ratio, chlorophyll content (*a* and *b*), leaf absorptance, maximum quantum efficiency of PSII in the dark-adapted state (*F*_v_/*F*_m_), and the light-saturated capacity of linear electron transport (ETRII) did not change compared with the wild type (Fig. 6I–M). Accordingly, light response curves of linear electron transport (Supplementary Fig. S13A), photoprotective non-photochemical quenching (qN, Supplementary Fig. S13B), the redox state of the PSII acceptor side (qL; Supplementary Fig. S13C), and the donor-side limitation of PSI (Y(ND); Supplementary Fig. S13D) also did not reveal significant differences between the wild type and the transplastomic lines. By contrast, the RNAi lines showed reduced chlorophyll content (to 27% of the wild-type levels in R1 and to 71% in R2), leaf absorptance (to 69% in R1, to 94% in R2, and to 95% in R3), *F*_v_/*F*_m_ (to 52% in R1, to 85% in R2, and to 96% in R3) and ETRII (to 29% in R1 and to 69% in R2; Fig. 6I–M). The light response curves of linear electron transport confirmed the strongly reduced electron transport capacity (Supplementary Fig. S13E). In the most strongly affected line, R1, induction of non-photochemical quenching was somewhat impaired (Supplementary Fig. S13F), in line with reduced contents of the xanthophyll cycle pigments violaxanthin and zeaxanthin (Fig. 3D, E). The redox state of the PSII acceptor side (Supplementary Fig. S13G) was less affected, but PSI became more rapidly oxidized in limited light (Supplementary Fig. S13H), in line with the strongly decreased capacity of linear electron transport.

### Primary/secondary metabolites are altered in transplastomic *DcLCYB1* and *NtLCYB* RNAi lines

**Fig. 7.**
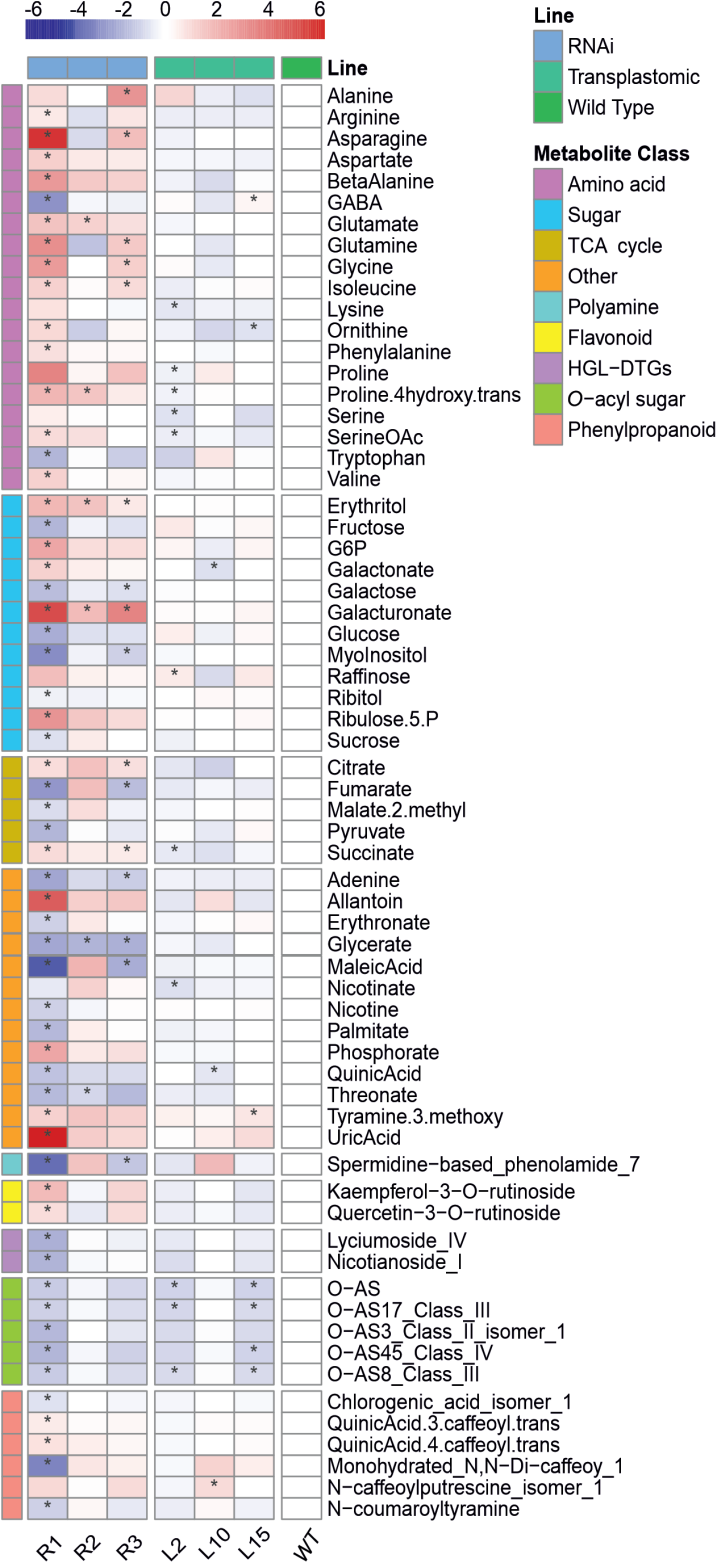
Metabolite content in transplastomic *DcLCYB1* and *NtLCYB* RNAi lines. Heat map of metabolite content for transplastomic and RNAi lines. Only metabolites with significant changes for at least one line are shown. Results are reported as log_2_-fold changes relative to the wild type. Statistical analysis was performed in R (R Core Team, 2018) using an unpaired Wilcoxon test with the default parameters within the function compare_means of the ggpubr package (Kassambara, 2018). WT, wild type.

To further investigate causes for the phenotype observed in the transgenic lines, metabolite levels were determined (see Materials and methods). A total of 72 primary (by GC-MS) and 31 secondary (by LC-MS) metabolites were measured. We observed 65 significant changes in *DcLCYB1* transplastomic and *NtLCYB* RNAi lines (Fig. 7). In the transplastomic lines only a few metabolites changed significantly (e.g. proline and serine for line L2). Additionally, three and four *O*-acyl sugars in line L2 and line L15 were decreased, respectively. However, other metabolites exhibited only a few or very moderate changes (Fig. 7). By contrast, the RNAi lines, especially the strongest variegated line, R1, exhibited extensive significant changes. Most of the amino acids were increased; for instance asparagine content increased by ~50-fold and glutamine content by ~8-fold in R1. The only amino acids that were decreased in R1 were GABA (~5-fold decrease) and tryptophan (~3-fold decrease). Furthermore, the abundance of most measured sugars was affected. Erythritol (~4-fold, ~3-fold, and ~2-fold increase for R1, R2, and R3, respectively) and galacturonate (~34-fold, ~3-, and ~11-fold increased for R1, R2, and R3, respectively) were increased in all three RNAi lines (Fig. 7). In the strongest variegated line, increased abundance of five sugars (e.g. galactonate ~2-fold increase) and decreased abundance of six sugars (e.g. *myo*-inositol ~6-fold decrease) was observed. Moreover, metabolites involved in the TCA cycle were reduced in R1 (e.g. fumarate and pyruvate with ~5- and ~3-fold decrease, respectively). Additionally, *O*-acyl sugars were significantly reduced in the R1 line (e.g. *O*-AS45 class IV ~3-fold reduction) and exhibited a similar trend for R3 (non-significant changes). Three phenylpropanoids were reduced (e.g. monohydrated *N*,*N*-dicaffeoyl-spermidine ~7-fold decrease) and two were increased (e.g. 4-*O*-*trans*-caffeoyl-D-quinic acid ~2-fold increase) in R1. Furthermore, allantoin and uric acid were increased ~25- and ~70-fold, whereas maleic acid and spermidine-based phenolamide #7 were ~14- and 10-fold decreased in R1 (Fig. 7). Altogether, the results suggest mild metabolic changes (primary and secondary) for transplastomic *DcLCYB1* lines but very pronounced metabolic changes in the line with the highest reduction in *NtLCYB* expression.

## Discussion

**Fig. 8.**
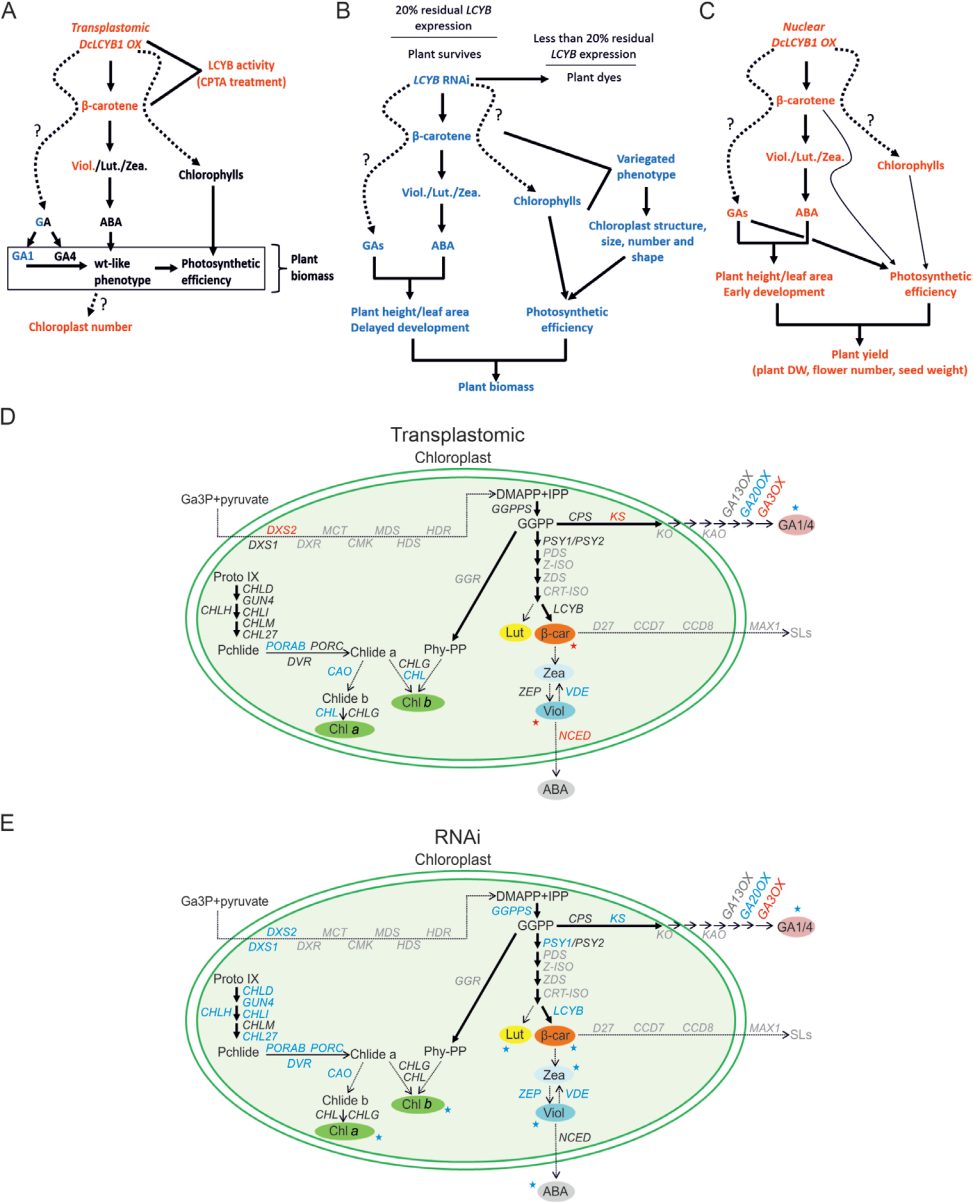
Proposed models for altered plant yield in transplastomic *DcLCYB1* and *NtLCYB* RNAi lines. (A) Proposed model for transplastomic lines. (B) Proposed model for RNAi lines. (C) Model for nuclear *DcLCYB1*-expressing lines (model was built from pigment, hormone, photosynthetic, and physiological data in Moreno *et al.* (2020)). In (A–C) the relations of physiological parameters (e.g. biomass, photosynthetic efficiency), gene expression, pigment content, and hormone content are shown (red font: increase; blue font: decrease; black font: no change). (D, E) Molecular response triggered by increased and reduced *LCYB* expression in transplastomic and RNAi lines, respectively. Changes occurring in at least two lines are shown. Increases are marked in blue, decreases in red. Genes shown in black were not changed and genes shown in grey were not measured. ABA, abscisic acid; β-car, β-carotene; Chl, chlorophyll; DMAPP, dimethylallyl diphosphate; GA, gibberellins; IPP, isopentenyl diphosphate; Lut, lutein; OX, overexpressor; Pchlide, protochlorophillidae; Phy-PP, phytyl diphosphate; SLs, strigolactones; Viol, violaxanthin; Zea, zeaxanthin. Gene abbreviation, protein name, and a brief description of each gene measured by qPCR can be found in Moreno *et al.* (2020).

Increased plant yield and photosynthetic efficiency from nuclear *DcLCYB1* expression was recently reported in tobacco (cv. Xanthi) (Moreno *et al.*, 2020). An increased GA/ABA ratio played a key role in the higher growth phenotype in the nuclear *DcLCYB1* tobacco lines (Fig. 8C). In an attempt to fully understand this phenomenon, we generated transgenic lines with high and reduced *LCYB* expression levels. Here, we took advantage of the high expression capacity granted by the transformation of plastid DNA and we generated transplastomic *DcLCYB1* tobaccos with very high *DcLCYB1* expression levels. High *LCYB* expression levels favored the β-branch of the carotenoid pathway, resulting in increased β-carotene and violaxanthin content. Interestingly, differences in *DcLCYB1* transcript levels within the transplastomic lines are not reflected in different levels of β-carotene accumulation. This has also been observed in our previous tobacco nuclear lines where L14 (~900-fold), L15 (~100-fold), and L16 (~60-fold) showed different *DcLCYB1* transcript levels but a similar β-carotene content increase of 2-fold (Moreno *et al.*, 2020). In carrot, this phenomena was observed in different tissues (leaf and root; Moreno *et al.*, 2013), where higher *DcLCYB1* expression in lines L6, L8, and L9 (2-, 4-, and 8-fold, respectively, compared with the wild type) was reflected in ~3-fold increase in β-carotene content. In carrot roots, different *DcLCYB1* transcript levels (1.25-, 1.6-, and 2.3-fold) was reflected in ~2-fold increase in β-carotene content. Altogether, these lines of evidence suggest that despite higher *DcLCYB1* transcript accumulation in the different transgenic lines, β-carotene accumulates at the same level in tobacco and carrot. This points towards the existence of an upper threshold in *LCYB* transcript levels and the accumulation of β-carotene.

Despite the extremely high *DcLCYB1* expression, transplastomic lines showed a wild type-like phenotype (Fig. 2A; Supplementary Fig. S1A–D). One possibility to explain the wild type-like biomass phenotype observed in the transplastomic lines lies in the connection between several isoprenoid plastid pathways. Carotenoid, chlorophyll, and GA biosynthesis pathways take place in the chloroplast and all share the same precursor, GGPP (Bouvier *et al.*, 2005; Pulido *et al.*, 2012). A positive co-expression of key genes encoding enzymes involved in the MEP, chlorophyll, carotenoid, and GA pathways accompanied by increased carotenoid, chlorophylls, and ABA/GA contents was observed in nuclear *DcLCYB1* tobaccos (Moreno *et al.*, 2020). By contrast, in our transplastomic lines we observed an altered/not consistent accumulation of transcripts encoding enzymes involved in MEP, tetrapyrrole, chlorophyll, LHCs, carotenoid, and ABA/GA biosynthesis pathways (Fig. 2H–L). For instance, GA20ox and GA3ox are key regulators of GA biosynthesis (Hedden and Thomas, 2012; Gupta and Chakrabarty, 2013). Increased *GA20ox* expression levels resulted in larger plants, higher GA content, biomass, and photosynthetic efficiency (Biemelt *et al.*, 2004; Voorend *et al.*, 2016). Gene expression of *GA20ox* was decreased in the transplastomic lines, whereas *GA3ox* expression was increased. This expression pattern is in line with the reduced GA_1_ and unchanged GA_4_ content in the transplastomic lines and might explain the observed wild type-like phenotype, in terms of biomass and photosynthetic efficiency (Figs 4A, B, 6I–M; Supplementary Figs S13A–D, S14A). Moreover, a reduced GA_1_ content might suggest a reduction in the GGPP pool as previously shown for carotenoid and chlorophyll contents in plants with modifications (*GGPPS* and *LCYB* overexpressors or the expression of an astaxanthin pathway) in the isoprenoid or carotenoid metabolic flux (Wurbs *et al.*, 2007; Apel and Bock, 2009; Tata *et al*., 2016; Lu *et al.*, 2017). Additionally, unchanged/moderately increased ABA content (L2) was not reflected in any developmental phenotype (Figs 4C, E, 5C, E). However, an ABA catabolite, phaseic acid (PA), has been shown to be a signaling molecule that fine-tunes plant physiology (e.g. seed germination, plant growth), environmental adaptation, and development (Weng *et al.*, 2016). That PA functions as a phytohormone (PA is recognized by a subset of ABA receptors) suggest that chemically related metabolites generated from plant hormones might additionally have signaling functions (Weng *et al.*, 2016), and therefore that it is important to analyse global changes in hormone metabolism caused by genetic manipulation of their precursors (e.g. ABA and GA). In our transplastomic lines, PA is reduced in L10 and L15 (Supplementary Fig. S7A) indicating that although ABA content is not drastically changed, ABA function might be influenced by the reduction in PA. In contrast, in our previously published nuclear *DcLCYB1* lines, the content of ABA and its catabolites (PA, neoPA, and ABA-glucose ester) was increased in all the lines. This evidence might suggest that together the lower β-carotene accumulation (30% more than in the wild type) and altered gene expression in the transplastomic lines (Fig. 2) in comparison with the nuclear *DcLCYB1* lines, affected ABA catabolism in a negative manner (reduced PA). GA metabolism was also affected in the transplastomic lines. For instance, reduction in *GA20ox* expression levels was correlated with GA_53_ accumulation and reduction of downstream-hydroxylated precursors (GA_19_ and GA_20_) of GA_1_ (Supplementary Fig. S7E, F), thus explaining the reduced GA_1_ content in our transgenic lines (Fig. 5A). Interestingly, hydroxylated and non-hydroxylated GA_29_ and GA_51_ contents were increased in the transplastomic lines pointing towards an increased *GA2ox* expression; however, we did not analyse the expression of this gene since it is not involved in the generation of bioactive GAs. Exogenous GA_3_ and GA_4_ application increased plant biomass in the transplastomic lines, while ABA treatment reduced biomass accumulation as previously described (Nagel *et al.*, 1994; Ma *et al.*, 2008). As previously reported, an appropriate GA/ABA ratio increases plant biomass in tobacco cv. Xanthi (Moreno *et al.*, 2020). Exogenous GA/ABA application (as previously reported) did not increase plant biomass in the transplastomic lines, probably due to the preexistent imbalance in ABA and GA metabolism in the transplastomic lines (Fig. 5A; Supplementary Fig. S7A–C). Taken together, our results suggest that very high *DcLCYB1* expression levels (i) shift the metabolic flux towards the β-branch of the carotenoid pathway, (ii) cause an altered expression pattern in isoprenoid genes of different plastid isoprenoid biosynthesis pathways, and (iii) (subsequently) influence phytohormone metabolism. In addition, increases in carotenoid content are not sufficiently high to alter the synthesis of apocarotenoids that have previously been reported to be involved in promoting growth (e.g. β-cc and zaxinone; Supplementary Fig. S10), which is in line with the wild type-like observed phenotype in the transplastomic lines.

Another possible explanation for the wild type-like phenotype observed in the transplastomic lines (Fig. 8A), could be related to a secondary effect from the plastid transformation. In transplastomic overexpressors, mRNA degradation or side products with lower molecular mass can accumulate and interfere with the accumulation of a desired metabolite (Wurbs *et al.*, 2007; Fernández-San Millán *et al*., 2018). For instance, plastid expression of *LCYB* genes from *Phycomyces blakesleeanus* (LP_P_) and *Erwinia* (LC_E_) did not influence carotenoid and chlorophyll content or plant growth in tobacco cv. Petit Havana (Wurbs *et al.*, 2007). However, LC_E_ did strongly increased CPTA tolerance, suggesting strong LCYB activity in these lines. In tomato, however, LC_E_ fruits produced a 4-fold increase in β-carotene content. Interestingly, in tomato leaves, β-carotene remain unchanged while reductions (lutein) and increases (antheraxanthin, zeaxanthin, and violaxanthin) of several carotenoids were observed. The unsuccessful expression of the LP_P_ was attributed to the observed instability of the transcript in the plastid. This may have occurred in our transplastomic lines, which accumulated lower levels of β-carotene compared with nuclear lines (Moreno *et al.*, 2020), despite higher steady state transcript accumulation of LYCB. However, in our transplastomic lines, the 30% increase in β-carotene (and ~50–200% in violaxanthin) content and high tolerance to CPTA suggest increased LCYB activity arguing against protein instability or enzymatic inactivity of the protein. In fact, to date, the 30% increase in β-carotene in our transplastomic lines is the highest reported in leaves of transplastomic *LCYB*-expressing tobacco and tomato lines (Wurbs *et al.*, 2007; Apel and Bock, 2009). Moreover, violaxanthin increase in leaves is higher in our transplastomic tobaccos than in leaves of transplastomic tomatoes (Wurbs *et al.*, 2007; Apel and Bock, 2009). This suggests that the *DcLCYB1* gene might confer an extra advantage for carotenoid production (due to different regulation or less susceptibility to feedback inhibition) compared with other *LCYB*s from daffodil and *Erwinia*. This might be explained by the vast β-carotene accumulation in leaves, but especially in carrot roots (Just *et al*., 2007, 2009). Interestingly, nuclear *DcLCYB1* overexpression in carrot increased 3-fold the β-carotene content in leaves (Moreno *et al.*, 2013), which is, to date, the highest increase in β-carotene content in leaves compared with previously reported nuclear and transplastomic transformations (Rosati *et al.*, 2000; Wurbs *et al.*, 2007; Apel and Bock, 2009; Ji *et al.*, 2009; Shi *et al.*, 2015). However, if we compared the transplastomic strategy with the nuclear transformation approach, it seems that the nuclear transformation approach is more efficient in terms of β-carotene accumulation. In our nuclear *DcLCYB1* lines (cv. Xanthi) (Moreno *et al.*, 2020), β-carotene accumulation is higher than in the transplastomic *DcLCYB1* lines (cv. Petit Havana, this work). In addition, fruits of transplastomic tomato plants expressing the daffodil *LCYB* produced less β-carotene than the fruits of the nuclear *LCYB* overexpressors (D’Ambrosio *et al.*, 2004; Apel and Bock, 2009). These lines of evidence show that in tobacco and tomato the nuclear strategy worked better than the transplastomic approach in terms of β-carotene accumulation. Although in the particular case of the *LCYB* expression in tobacco and tomato the nuclear strategy was proven to be more efficient, the insertion of an astaxanthin pathway by a transplastomic approach resulted in pink/orange tobacco plants (Lu *et al.*, 2017), due to an extremely high astaxanthin accumulation. This suggests that strategies to manipulate carotenoid genes and enhance carotenoid content in plants have to be evaluated case by case.

By contrast, the reduced *NtLCYB* expression caused leaf variegation in the RNAi lines, as reported for other mutants in Arabidopsis, *Brassica napus*, and rice, with defects in carotenoid biosynthesis (Aluru *et al*., 2001, 2009; Fang *et al.*, 2008; Zhao *et al.*, 2020). Here, by choosing three lines with different phenotype strength and gene expression we were able to dissect the importance of β-carotene for plant growth and development. Although strong *LCYB* RNAi lines (R1 and R2) showed strong plant variegation and perhaps accumulation of secondary effects not related to the *LCYB* silencing, the phenotype of wild type CPTA-treated plants mimics the variegated and reduced growth phenotype observed in the stronger RNAi lines (Supplementary Fig. S1). Although we do not exclude secondary effects, this suggests that in these RNAi lines most of the observed molecular and phenotypic effects might derive from the *LCYB* silencing. Interestingly, a similar variegated phenotype was reported for tobacco plants with reduced *LCYB* expression, but the lack of detailed gene expression analysis, chloroplast structure, and phytohormone and photosynthesis measurements impedes the ability to draw strong conclusions about the origin of this phenotype at the molecular and physiological levels (Shi *et al.*, 2015). Here, we found that *LCYB* expression is essential for plant autotrophic growth (Fig. 8B; Supplementary Fig. S3). Residual *LCYB* expression of 20% (equivalent to 35% residual levels of β-carotene and other derivatives) or higher is nonetheless sufficient for survival in plants germinated aseptically and transferred to soil, while we deduce that less than 20% *LCYB* expression/35% β-carotene content (and other carotenoids) is not enough for plants to survive when grown on soil (Fig. 8B; Supplementary Fig. S3A–C).

Reduced *LCYB* expression caused the opposite effect observed in the nuclear *DcLCYB1* tobacco lines (Moreno *et al.*, 2020), characterized by a down-regulation of key genes involved in the MEP (*DXS*), chlorophyll (*CAO*, *DVR*, *PORC*, and *PORAB*), carotenoid (*PSY1* and *LCYB*), and GA (*KS* and *GA20oxidase*) pathways accompanied by decreased carotenoid, chlorophylls and ABA/GA contents, thus suggesting a depletion of the common GA, chlorophylls, and carotenoid pathway precursor GGPP in the strongest RNAi lines, which is in line with the reduction in chlorophyll, carotenoid, and GA contents in an Arabidopsis *pds* mutant (Qin *et al.*, 2007). This idea is supported by the strong down-regulation of key genes from carotenoid and MEP pathways (*PSY1*, *DXS1*, and *DXS2*), which are part of a feedback mechanism, known to control the content of carotenoids and their precursors in Arabidopsis (Carretero-Paulet *et al.*, 2006; Cazzonelli and Pogson, 2010; Ruiz-Sola and Rodríguez-Concepción, 2012). These lines of evidence (together with the nuclear and transplastomic *DcLCYB1*-expressing lines) suggest the possibility of the existence of hotspots in the carotenoid pathway that could trigger positive or negative feedback loops to communicate with other isoprenoid pathways. Further proof of these feedback mechanisms is the strong reduction in transcript accumulation of *GGPPS*, which encodes GGPP SYNTHASE, an enzyme that catalyses the conversion of isopentenyl diphosphate/dimethylallyl diphosphate into GGPP (Bouvier *et al.*, 2005; Pulido *et al.*, 2012). This reduction is in line with the reduced GA, chlorophyll, and carotenoid content.

Several molecular and phenotypic characteristics of the RNAi lines are reminiscent of plants with reduced ABA and GA content (Schomburg *et al.*, 2003; Nitsch *et al.*, 2012). In fact, the early seed germination, reduced biomass and leaf area, and reduced primary root growth observed in the strongest RNAi lines might reflect reduced ABA and GA content (Fig. 5C–E; Supplementary Figs S4B, S5E–H) (Schomburg *et al.*, 2003; Nitsch *et al.*, 2012). In addition, decreased ABA function and content might be reflected in reductions in the content of the ABA-derived phytohormone PA (Weng *et al.*, 2016) and the ABA-glucose ester (which serve as ABA storage). Interestingly, exogenous GA_3_, GA_4_, ABA, GA_3_/ABA, and GA_4_/ABA application in the RNAi lines did not rescue the phenotype to the wild type level (Fig. 5G), suggesting the involvement of other factor(s) explaining the reduced biomass phenotype in our RNAi lines. In fact, the plant variegation (leaves, stem, and capsules) observed in the strongest RNAi lines (Supplementary Fig. S4B–D) points towards pigment, photosynthesis, and chloroplast biogenesis defects in these lines. Moreover, other carotenoid-defective mutants, and specially *pds3* mutants, showed impaired chloroplast differentiation and development, and reduced chloroplast number (Aluru *et al.*, 2001; Qin *et al.*, 2007; Zhao *et al.*, 2020). Carotenoids are located in the photosynthetic membrane in the form of chlorophyll–carotenoid–protein complexes and some carotenogenic enzymes are membrane-associated (Green and Durnford, 1996; Cunningham and Gantt, 1998). In this way, any changes in carotenoid composition always lead to abnormal plastid development (Welsch *et al.*, 2000; Park *et al.*, 2002). Recently, the expression of a bacterial *CrtB* (*PSY*) gene (in Arabidopsis, lettuce, tobacco, and zucchini), and as a consequence of that, a burst in phytoene accumulation, was shown to stimulate differentiation of chloroplast into chromoplast suggesting that indeed carotenoid content influences chloroplast development (Llorente *et al.*, 2020). Interestingly, we observed increased chloroplast number in our transplastomic tobacco lines (Supplementary Table S4), which is in line with the increased chromoplast number observed in tomato fruits expressing the citrus *LCYB* gene (Zhu *et al.*, 2020). Taken together this evidence suggests a connection between carotenoid content and chloroplast biogenesis and development that deserves further attention.

Carotenoids participate in photoprotection mechanisms by (i) modulating the non-radiative dissipation of excess excitation energy (Niyogi *et al.*, 1998; Dall’Osto *et al.*, 2005), (ii) mediating direct quenching of chlorophyll (Chl) triplets (^3^Chl*), or (iii) scavenging ROS generated during photosynthesis (Niyogi, 2000; Havaux *et al.*, 2004; Dall’Osto *et al.*, 2005, 2007*a*). In addition, carotenoids are structural determinants of the photosynthetic apparatus (Cazzaniga *et al.*, 2012), and only a minor fraction is free in the lipid phase of thylakoids where it serves as an antioxidant (Havaux *et al.*, 2004) and modulates the fluidity of the lipid bilayer (Gruszecki and Strzałka, 2005). For instance, β-carotene is bound to reaction center subunits of both PSI and PSII, whereas xanthophylls are bound to peripheral LHC subunits that comprise the antenna system (Cazzaniga *et al.*, 2012). Thus, it is expected that any disturbance of carotenoid content can be reflected in alterations of carotenoid function but also in the biogenesis of several components of the photosynthetic apparatus. The Arabidopsis *szl1* mutant, which carries a point mutation in the *LCYB* gene, and thus exhibits a less-active LCYB enzyme, showed reduced β-carotene (55% of the wild type), violaxanthin, and zeaxanthin, increased lutein accumulation (2-fold), and slightly reduced growth (Li *et al.*, 2009; Cazzaniga *et al.*, 2012). Reductions in these pigments caused reduced *F*_v_/*F*_m_, chlorophyll content, qE (the rapidly reversible component of NPQ), and increased photooxidation (higher ^1^O_2_ production). In our stronger RNAi lines, β-carotene content was similarly reduced (55–60%), but lutein, zeaxanthin, and violaxanthin were reduced, causing strong reductions in chlorophyll content (up to 67%), *F*_v_/*F*_m_ (up to 50%), ETRII, and qN, affecting strongly plant photosynthetic performance (Fig. 6I–M; Supplementary Fig. S13E–H). In addition, strong reduction in β-carotene and xanthophyll contents impact on thylakoid membrane integrity and the assembly and stability of the photosynthetic complexes. For instance, lutein is required for the folding of LHC proteins (Formaggio *et al.*, 2001) and zeaxanthin and violaxanthin bind sites L2 and/or V1 in LHCB proteins to increase resistance to high light and exogenously provided photosensitizers (Baroli *et al.*, 2004) by scavenging ^1^O_2_ and thus preventing lipid peroxidation (Havaux and Niyogi, 1999; Havaux *et al.*, 2007). Reduced xanthophyll and β-carotene are also reflected in higher accumulation of ^1^O_2_ in our RNAi lines and other carotenoid mutants (Li *et al.*, 2009; Cazzaniga *et al.*, 2012). Alteration in ROS and ROS-processing systems has been reported to modulate plant growth and development (Pagnussat *et al.*, 2005; Kirchsteiger *et al.*, 2012; Yu *et al.*, 2013; Mhamdi and Van Breusegem, 2018), and thus ROS accumulation in our RNAi lines might contribute to the observed reduced growth and delayed development. In addition, altered chloroplast ultrastructure (including chloroplast size, shape, and number; Fig. 6E–H; Supplementary Fig. S12E, F, K–N; Supplementary Table S4) and altered cellular level organization of the leaf in the yellow tissue of the RNAi lines (Supplementary Fig. 11E) might explain the reduced growth phenotype. In fact, altered chloroplast ultrastructure and development were observed in other mutants with reduced expression of other carotenoid genes (*PDS*, *ZDS*, *CRTISO*), with mutant lines also showing impaired or delayed growth (Aluru *et al*., 2001, 2009; Qin *et al.*, 2007; Fang *et al.*, 2008; Zhao *et al.*, 2020). In general, these results expose strong defects in plastid biogenesis (in yellow sectors of the leaves) in the RNAi lines, which are reflected in altered structure and function of the photosynthetic apparatus. The aforementioned evidence is in line with the massive reduction in amino acids, sugars, and TCA cycle intermediates in the strongest RNAi line (Fig. 7), as well as with the dramatic reductions in accumulation of transcripts with products required for LHC (Fig. 3J), PSI, cyt *b*_*6*_*f*, and PSII (Supplementary Figs S4C, D, S14B), and tetrapyrrole and chlorophyll biosynthesis processes (Fig. 3F, G, K, L). The generated evidence points towards three factors contributing to reduced biomass (Fig. 8E) in the RNAi lines: (i) reduced pigment content and photosynthetic efficiency, (ii) altered plastid biogenesis, and (iii) reduced phytohormone content. In addition, the strongest RNAi line (R1) showed a reduction in accumulation of zaxinone, a recently reported growth (promoting) regulator in rice (Wang *et al.*, 2019), suggesting that zaxinone might be contributing to the observed growth phenotype. However, zaxinone content was the same as the wild type for the R2 line, which showed a similar phenotype as R1, arguing against a zaxinone contribution to this phenotype.

In summary, manipulation of plastid DNA to increase *LCYB* expression levels showed that very high *DcLCYB1* expression, for the first time in transplastomic lines, results in significantly higher β-carotene content. However, it can also disturb the expression pattern of key genes of carotenoid and carotenoid-related biosynthesis pathways, impeding the accumulation of key end products (e.g. GA) from other isoprenoid pathways (Fig. 8A, D). Reduced (GA_1_) and unchanged (GA_4_ and ABA) phytohormone content are reflected in the wild type-like phenotype in the transplastomic lines (in contrast with the high yield phenotype observed in the nuclear lines (Moreno *et al.*, 2020); Fig. 8C). Strategies to modify metabolic fluxes in isoprenoid pathways through plastid transformation have to be analysed case by case, because as shown in our results higher gene expression will not always be reflected in higher levels of the desired metabolite. Reduction of *LCYB* expression affects several molecular events (Fig. 8E) that influence plant development and metabolism, and resulted in decreased photosynthetic efficiency and plant biomass (Fig. 8B).

Altogether, our previous work coupled with the current results, which combined nuclear, transplastomic, and RNAi lines (Fig. 8A–C), leads us to conclude that by manipulating *LCYB* expression it is possible to modulate plant biomass by modifying pigment and hormone content and other molecular processes within the plastid. Our data also suggest a threshold for *LCYB* expression levels that might be beneficial or detrimental for plant yield, which serve as a guideline to export this genetic engineering strategy into crops.

## Supplementary data

The following supplementary data are available at *JXB* online.

Fig. S1. Transplastomic and wild type plants exposed to CPTA (2-(4-chlorophenylthio) triethylamine) treatment.

Fig. S2. Relative expression of key genes comprising PSI, PSII, and cyt *b*_*6*_*f* complex.

Fig. S3. T_0_ RNAi lines growing in MS media and on soil.

Fig. S4. 16-week-old transplastomic and RNAi tobacco plants.

Fig. S5. Quantification of physiological parameters of *DcLCYB1* transplastomic and *NtLCYB* RNAi lines.

Fig. S6. T_3_ RNAi plants at different stages of their life cycle.

Fig. S7. ABA and GA metabolism in transplastomic *DcLCYB1* lines.

Fig. S8. ABA and GA metabolism in *NtLCYB* RNAi lines.

Fig. S9. Hormone and inhibitor treatments in transplastomic *DcLCYB1* and *NtLCYB* RNAi lines.

Fig. S10. Apocarotenoid quantification in transplastomic *DcLCYB1* and *NtLCYB* RNAi lines.

Fig. S11. Light microscopy images of leaf cross sections of transplastomic and RNAi lines.

Fig. S12. Transmission electron microscopy (TEM) images of tobacco cells from the *NtLCYB* RNAi line R1 and R3.

Fig. S13. Light response curves of photosynthetic parameters in transplastomic and RNAi lines.

Fig. S14. Schematic model representing changes in gene expression of photosystem I, II, and the cyt *b*_*6*_*f* complex subunits in transplastomic and RNAi lines.

Table S1. Primers used for qPCR experiments.

Table S2. In vitro regeneration process for the generation of *DcLCYB1* transplastomic tobacco plants.

Table S3. End-point measurement of plant height and flower number of 16-week-old *NtLCYB* RNAi lines.

Table S4. Chloroplast number per area in transplastomic *DcLCYB1* and *NtLCYB* RNAi lines.

Dataset S1. LC-MS metabolite-reporting list following the recommendations described by Fernie *et al.* 2011.

Dataset S2. GC-MS metabolite-reporting list following the recommendations described by Fernie *et al.* 2011.

Dataset S3. The SRM transition list for apocarotenoid profiling.

erab029_suppl_Supplementary_Datasets_S1-S3

erab029_suppl_Supplementary_Figures_S1-S14_Tables_S1-S4

## Data Availability

All relevant data can be found within the manuscript and its supplementary data.
