# Supplementary material for: Lycopene β-cyclase expression influences plant physiology, development, and metabolism in tobacco plants
Source: J Exp Bot. 2021 Jan 23;72(7):2544–69. doi: 10.1093/jxb/erab029 (PMC8006556; doi:10.1093/jxb/erab029)
Supplement: erab029_suppl_Supplementary_Figures_S1-S14_Tables_S1-S4 [file erab029_suppl_supplementary_figures_s1-s14_tables_s1-s4.pdf]

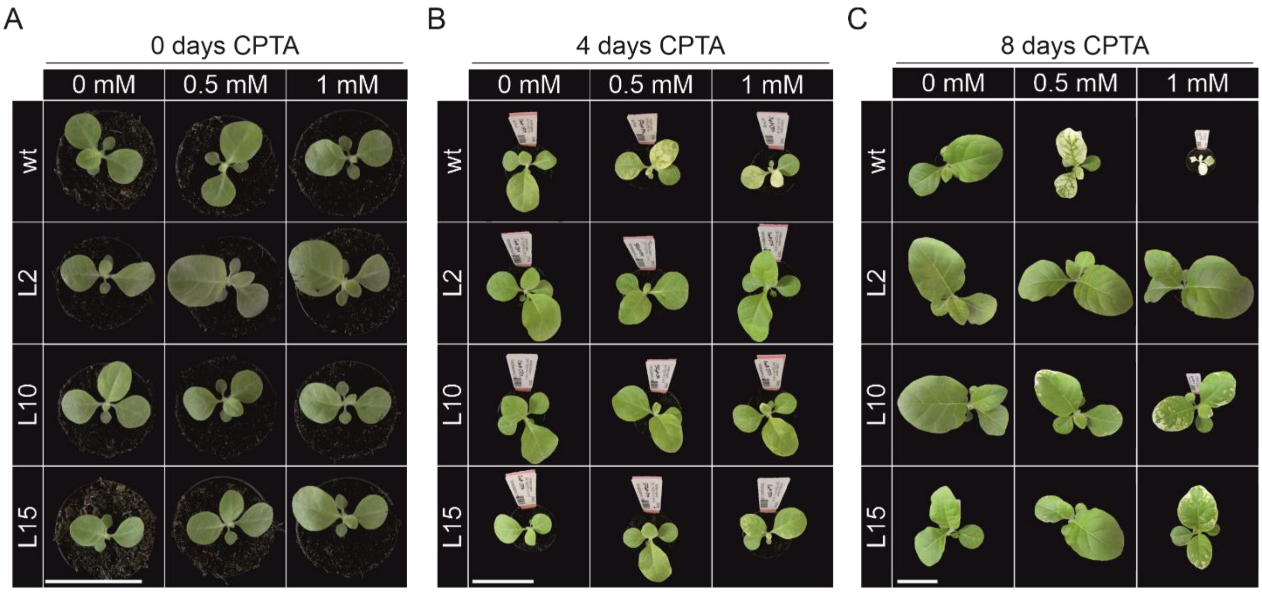

Supplementary Fig. S1. Transplastomic and wild type plants exposed to CPTA (2-(4-chlorophenylthio) triethylamine) treatment. Transplastomic (2-week old) lines L2, L10, and L15 and wild type were exposed to different concentrations of CPTA (0, 0.5 and 1 mM) for 8 days. A, After 0 days. B, After 4 days. C, After 8 days. CPTA tolerance is enhanced in transplastomic lines where line L2 remained green after eight days of 1 mM CPTA treatment, lines L10 and L15 showed a slight leaf variegation and reduced growth, and the wild type showed chlorosis and stunt growth. Three biological replicates were used per line and condition. wt: wild type, L2: pJM36-2, L10: pJM37-10, L15: pJM37-15, scale bar: 10 cm.

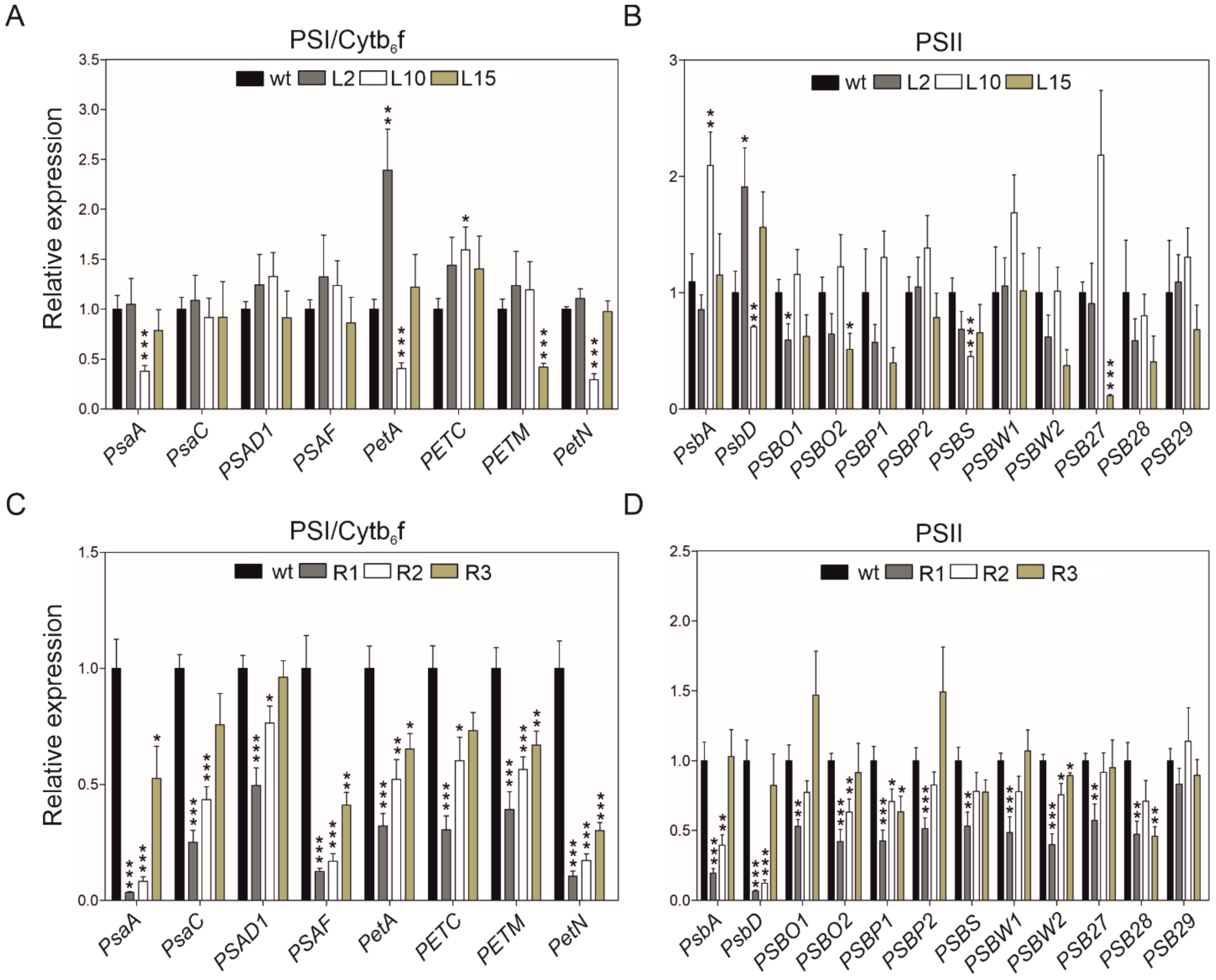

Supplementary Fig. S2. Relative expression of key genes comprising PSI, PSII and cyt b_6f complex. Stable accumulation of key PSI, PSII and cyt b_6f complex genes in *DcLCYB1* transplastomic lines (A and B) and *NtLCYB* RNAi lines (C and D) measured by qRT-PCR. A, C, Expression analysis of photosystem I and cyt b_6f complex subunits. B, D, Expression analysis of photosystem II subunits. Columns and bars represent the means and the ±SEM (three biological and three technical replicates). Non-paired two-tailed student t-test was performed to compare transgenic lines with the wild type. *: $p < 0.05$, **: $p < 0.001$, ***: $p < 0.0001$. PSI: photosystem I, PSII: photosystem II, Cyt b_6f: cytochrome b_6f complex, wt: wild type.

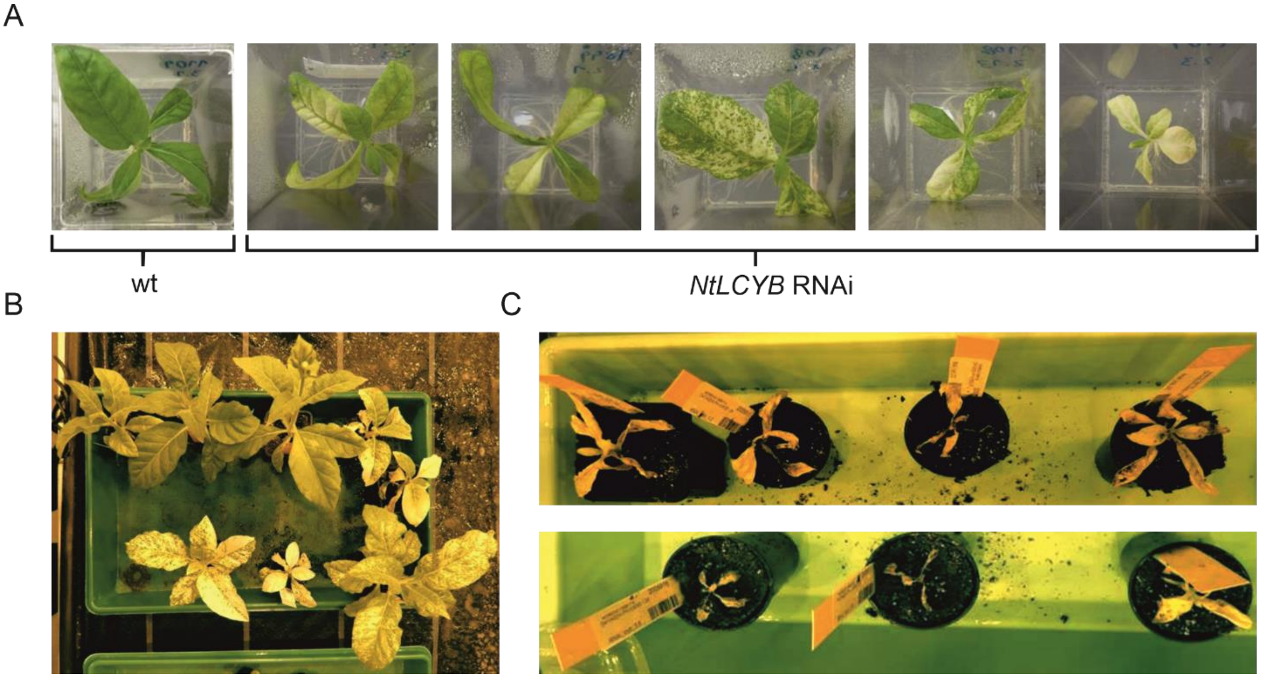

Supplementary Fig. S3. T0 RNAi lines growing in MS media and on soil. A, RNAi lines growing on MS media showing different degrees of variegation. B, *NtLCYB* RNAi lines (variegated/wild type-like phenotype) grown on soil showing different degrees of variegation. C, *NtLCYB* RNAi lines (white/pale leaf phenotype) after 3 and 1 week growing on MS media and soil, respectively. wt: wild type.

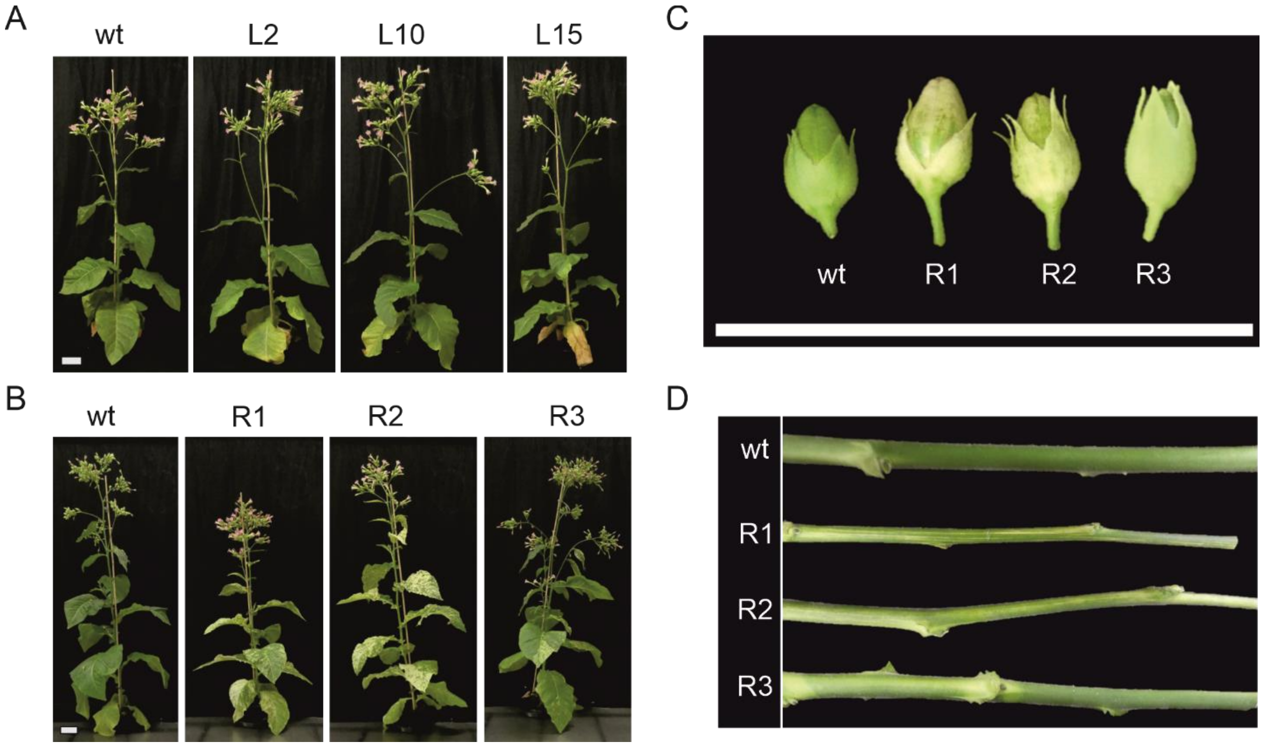

Supplementary Fig. S4. 16-week-old transplastomic and RNAi tobacco plants. A, T1 transplastomic *DcLCYB1* plants. B, T1 *NtLCYB* RNAi plants. C, capsules of RNAi lines. D, Stems of RNAi lines. wt: wild type, scale bar: 10 cm.

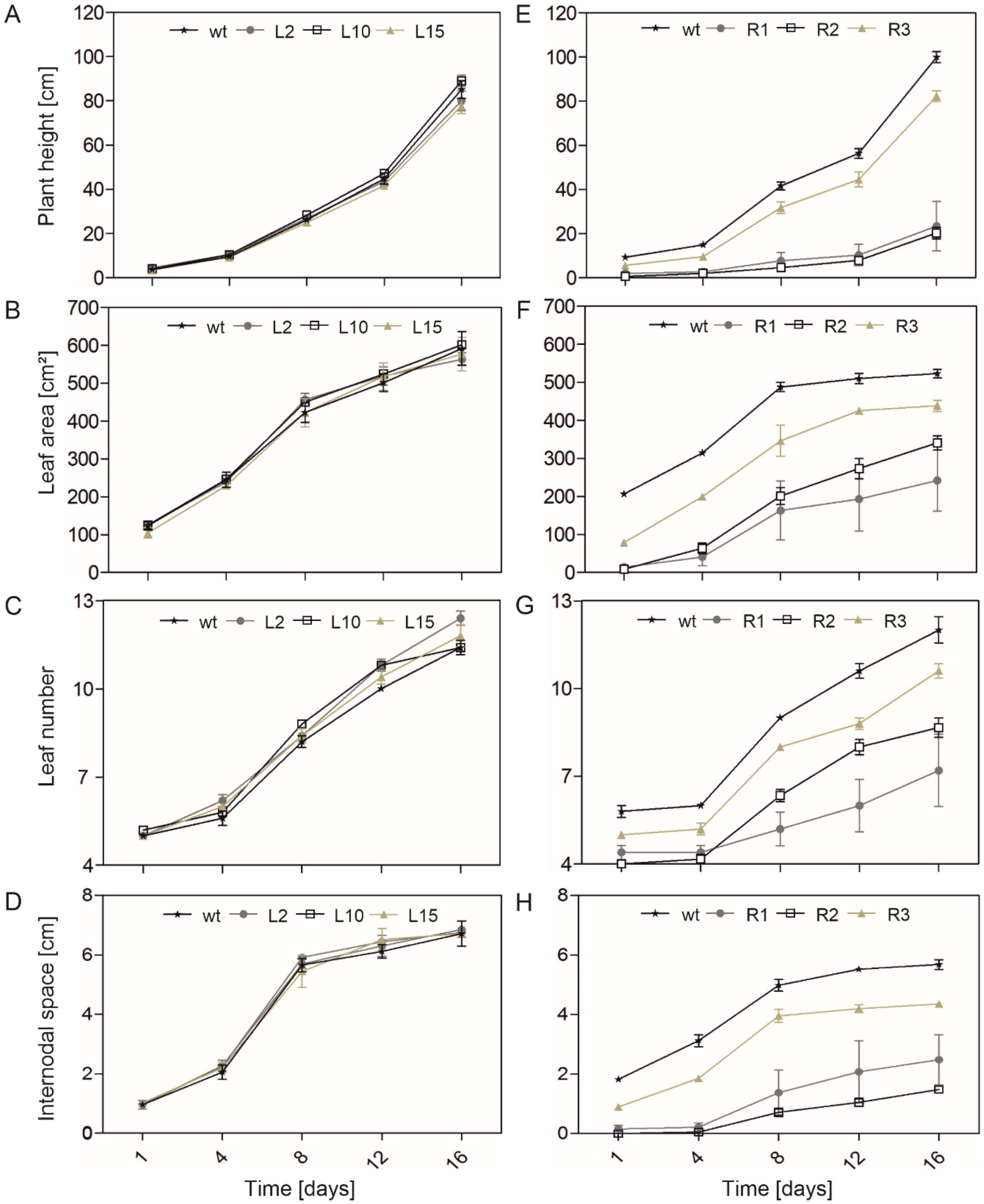

Supplementary Fig. S5. Quantification of physiological parameters of *DcLCYB1* transplastomic and *NtLCYB* RNAi lines. Plant height, leaf area, leaf number and internode space were analyzed at different time points during plant development of transplastomic (A-D) and RNAi lines (E-H). A, E, plant height. B, F, leaf area. C, G, leaf number. D, H, internodal space was measured in two consecutive leaves (4th and 5th).

All the physiological measurements were performed as described in Moreno et al., 2020. In transplastomic lines, no significant changes were observed with the exception of an increase in leaf number for line L2 at day 12 and 16, whereas in RNAi lines all investigated parameters were significantly reduced during the entire development. Non-paired two-tailed Student's t-test was perform to compare transgenic lines with the wild type. wt: wild type.

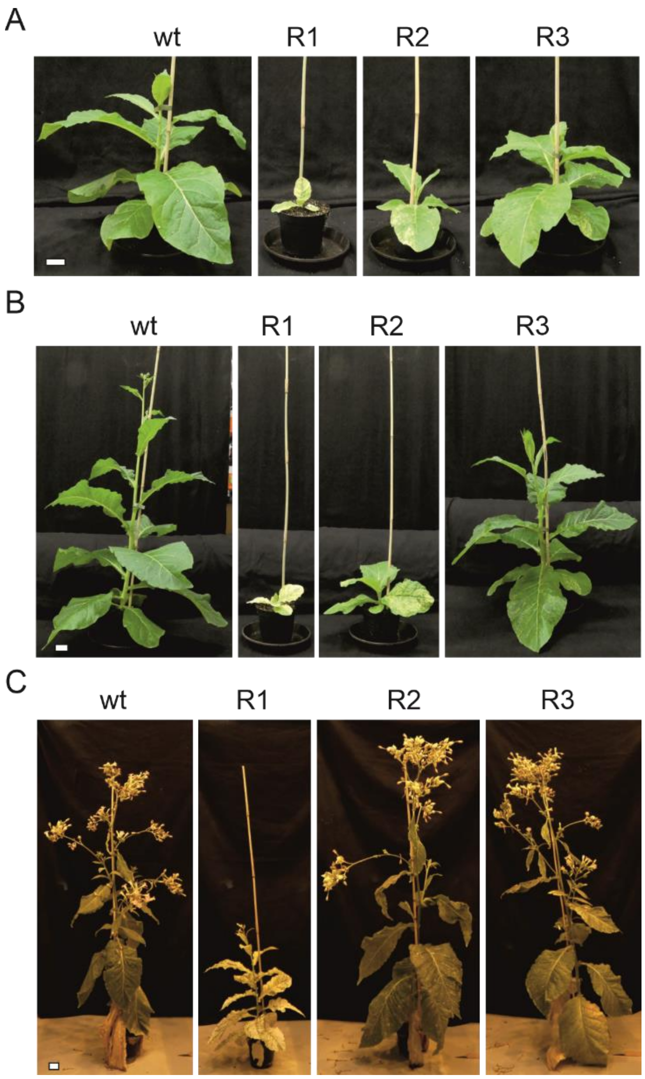

Supplementary Fig. S6. T3 RNAi plants at different stages of their life cycle. A, B, 8- and 9-week old wild type and T3 RNAi plants, RNAi lines exhibit different variegation strength. C, 16-week old wild type and T3 RNAi plants. At 16 weeks growth of R2 and R3 are similar to wt while R1 is still shorter. wt: wild type, scale bar: 10 cm.

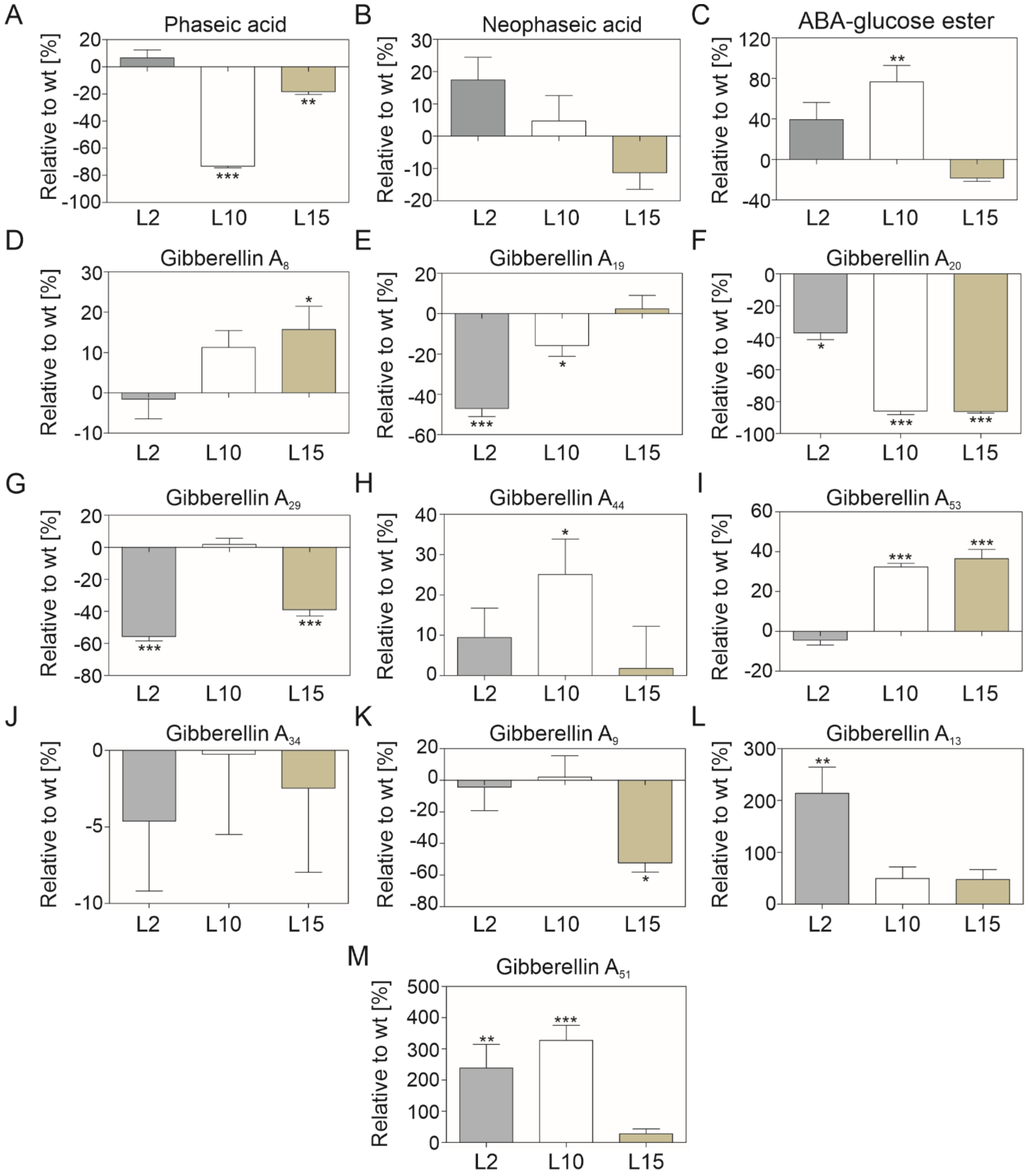

Supplementary Fig. S7. ABA and GA metabolism in transplastomic *DcLCYB1* lines. A-C, Contents of ABA catabolites (phaseic acid, neophaseic acid and ABA-glucose ester) expressed in percentage relative to the wild type (set to 100%) and measured by UPLC-ESI(-/+)-MS/MS, as described in the Materials and

Methods section. D-M, Contents of GA precursors and catabolites (GA_8, GA_9, GA_{13}, GA_{19}, $GA_{20,}$ GA_{29}, GA_{34}, GA_{44}, $GA_{51}$and GA_{53}) expressed in percentage relative to the wild type (set to 100%) and measured by UHPLC-(-ESI)-MS/MS. Columns and bars represent the means and the ±SEM of 5 biological replicates and three technical replicates. Unpaired Student's t-test was performed to compare transgenic lines with the wild type. *: $p < 0.05$, **: $p < 0.005$, ***: $p < 0.0005$. ABA: abscisic acid, GA: Gibberellins, wt: wild type.

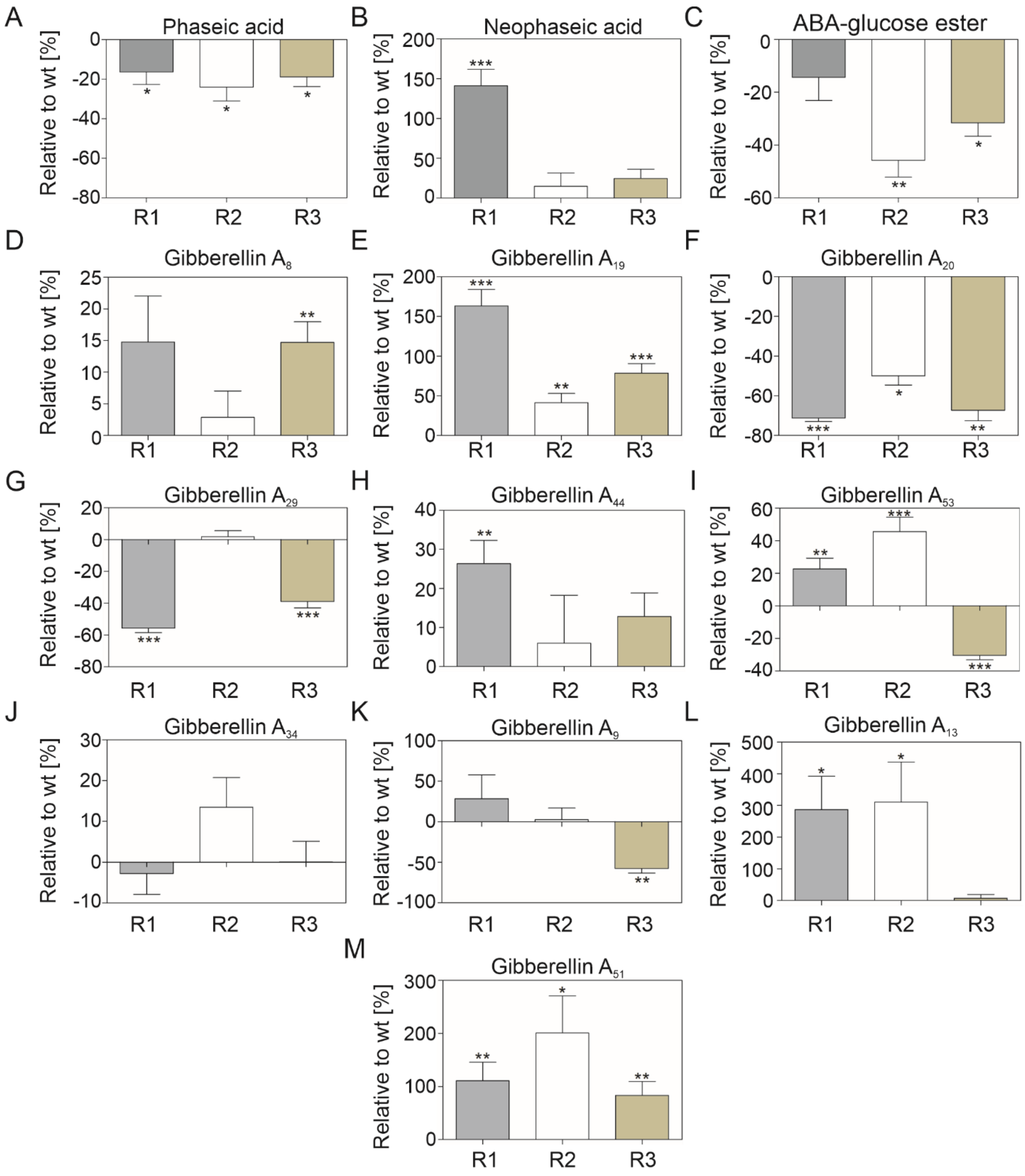

Supplementary Fig. S8. ABA and GA metabolism in *NtLCYB* RNAi lines. A-C, Contents of ABA catabolites (phaseic acid, neophaseic acid and ABA-glucose ester) expressed in percentage relative to the wild type (set to 100%) and measured by UPLC-ESI(-/+)-MS/MS, as described in the Materials and Methods section. D-M, Contents of GA precursors and catabolites (GA_8, GA_9, GA_{13}, GA_{19}, GA_{20}, GA_{29},

GA_{34}, GA_{44}, $GA_{51}$and GA_{53}) expressed in percentage relative to the wild type (set to 100%) and measured by UHPLC-(-ESI)-MS/MS. Columns and bars represent the means and the ±SEM of the 5 biological replicates and three technical replicates. Unpaired Student's t-test was performed to compare transgenic lines with the wild type. *: $p < 0.05$, **: $p < 0.005$, ***: $p < 0.0005$. ABA: abscisic acid, GA: Gibberellins, wt: wild type.

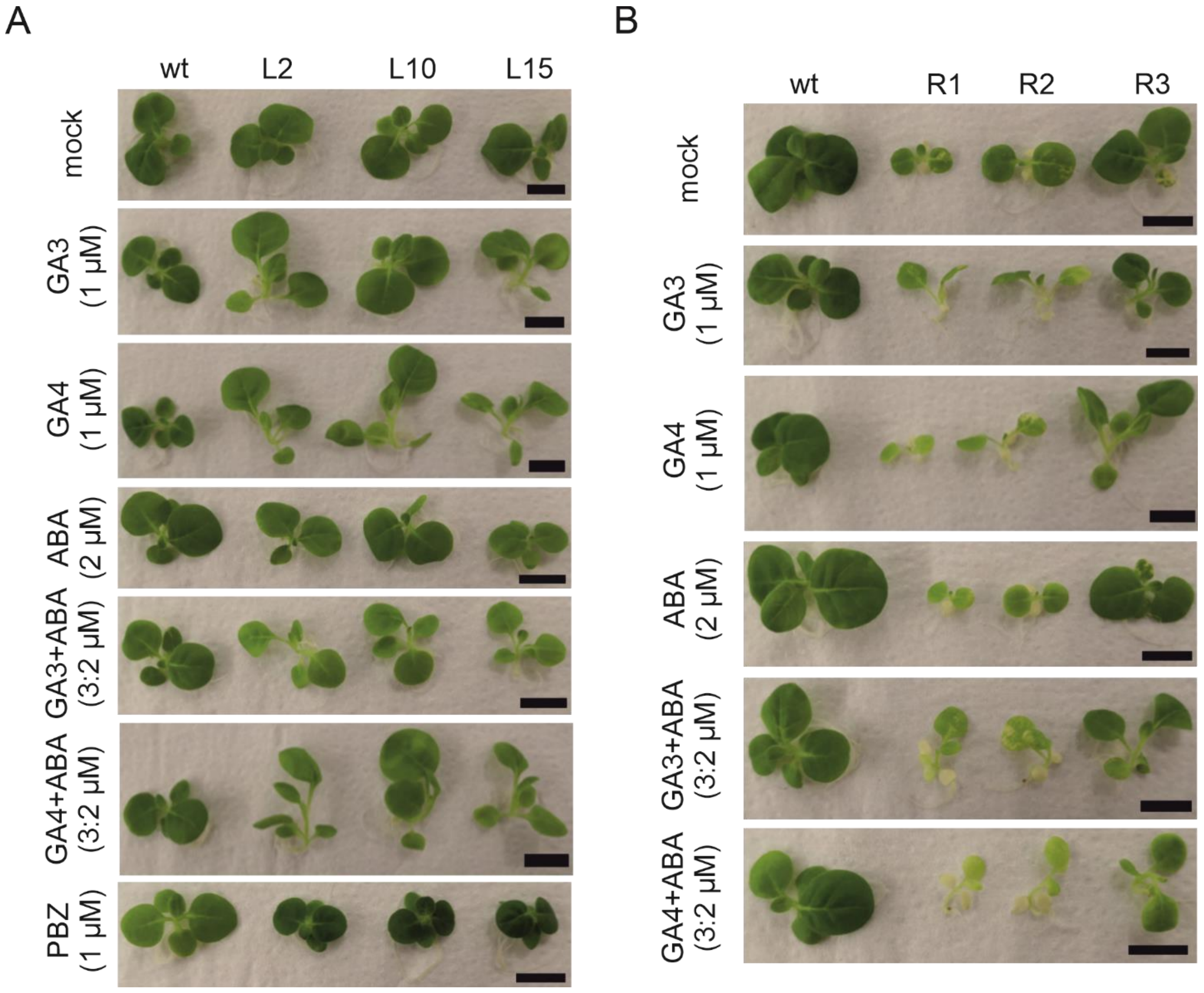

Supplementary Fig. S9. Hormone and inhibitor treatments in transplastomic *DcLCYB1* and *NtLCYB* RNAi lines. A, Phenotypes of *DcLCYB1* transplastomic lines. B, Phenotypes of *NtLCYB* RNAi lines. Tobacco seedlings were grown on agar media (1 % sucrose) for 10 days and subsequently transferred to liquid MS-media (1 % sucrose) supplemented with hormones and inhibitors. Phenotypes of 17-day old wild type, transplastomic and RNAi lines treated with H_2O (mock), GA3 (1 µM), GA4 (1 µM), ABA (2 µM), GA3/ABA (3 µM/2 µM), GA4/ABA (3 µM/2 µM), and paclobutrazol (PBZ, 1 µM) for seven days. Phenotypes were recorded after seven days of treatment. Scale bar: 10 cm.

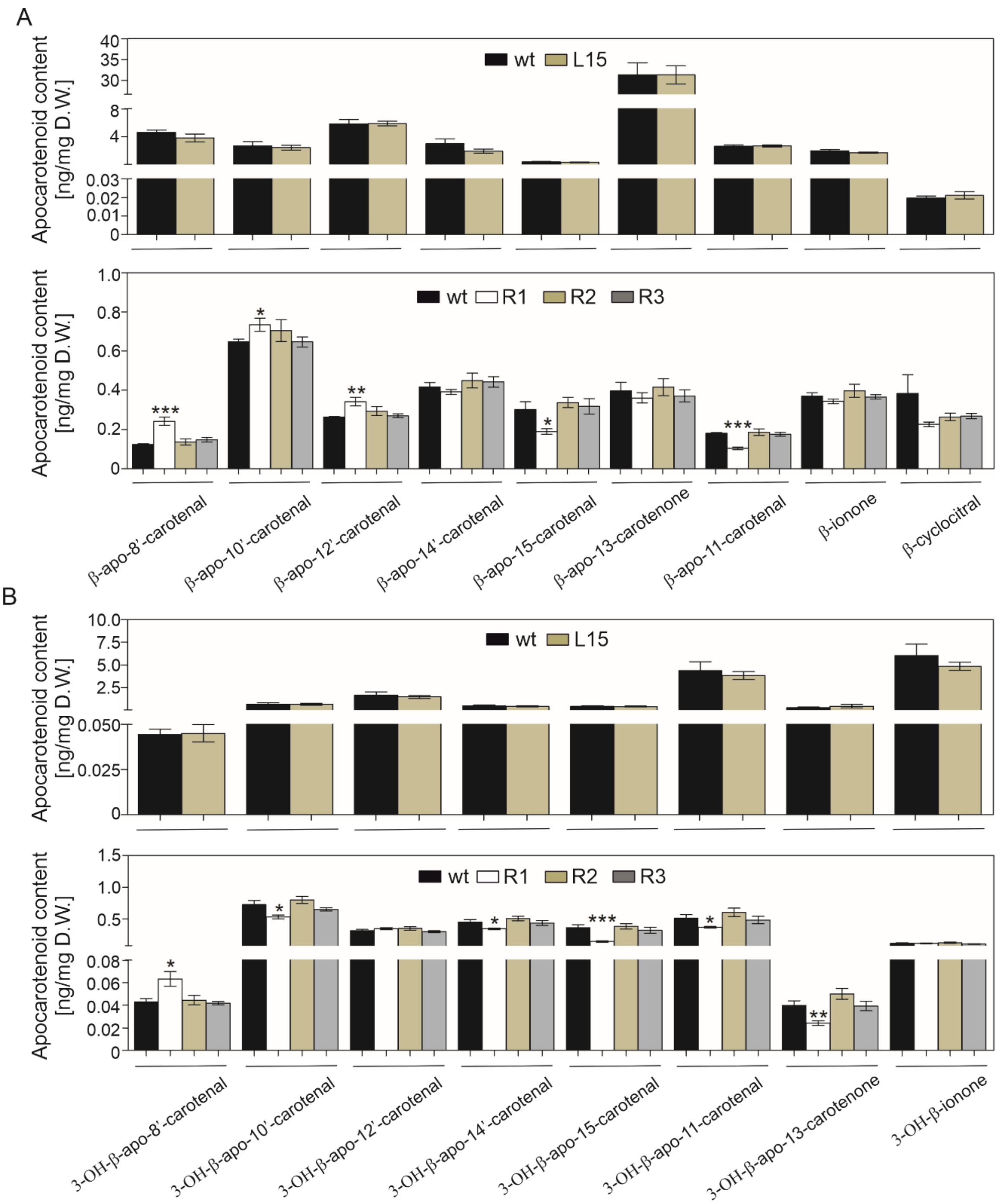

Supplementary Fig. S10. Apocarotenoid quantification in transplastomic *DcLCYB1* and *NtLCYB* RNAi lines. (A, B) Non-hydroxylated and hydroxylated apocarotenoid species were quantified in the 4th leaf from 5-week-old tobacco leaves of wild type and transgenic lines using an UHPLC-MS system. Columns and bars represent the means ±SEM (n = 6). Unpaired Student's t-test was performed to compare transgenic lines with the wild type. *: $p < 0.05$, **: $p < 0.01$; ***: $p < 0.005$.

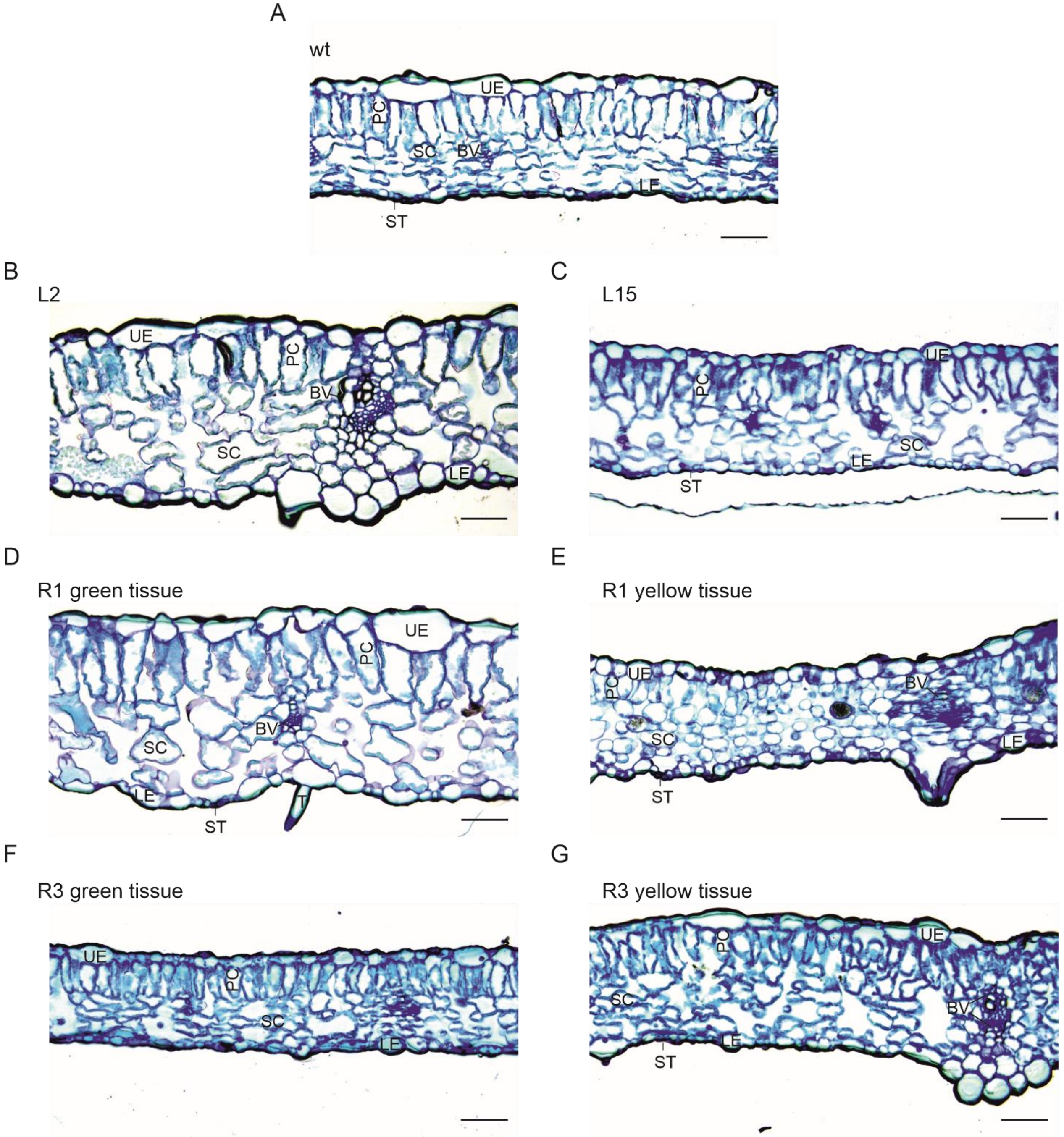

Supplementary Fig. S11. Light microscopy images of leaf cross sections of transplastomic and RNAi lines. A-C, in transplastomic lines no differences in the arrangement of cell layers was observed. D, F, G, in the green tissue of R1 and both green and yellow tissue of R3 no changes in cellular organization were observed. E, in contrast, in the yellow tissue of R1 leaf cross sections smaller palisade cells with reduced side-by-side orientations occur. Representative images of leaves from transplastomic and RNAi lines (n=4).

Sections were stained with 0.05% Toluidine blue. BV: bundle vessels, LE: lower epidermis, PC: palisade cell, SC: spongy mesophyll cell, ST: stoma, T: trichome, UE: upper epidermis, wt: wild type. Scale bar: 100 µm.

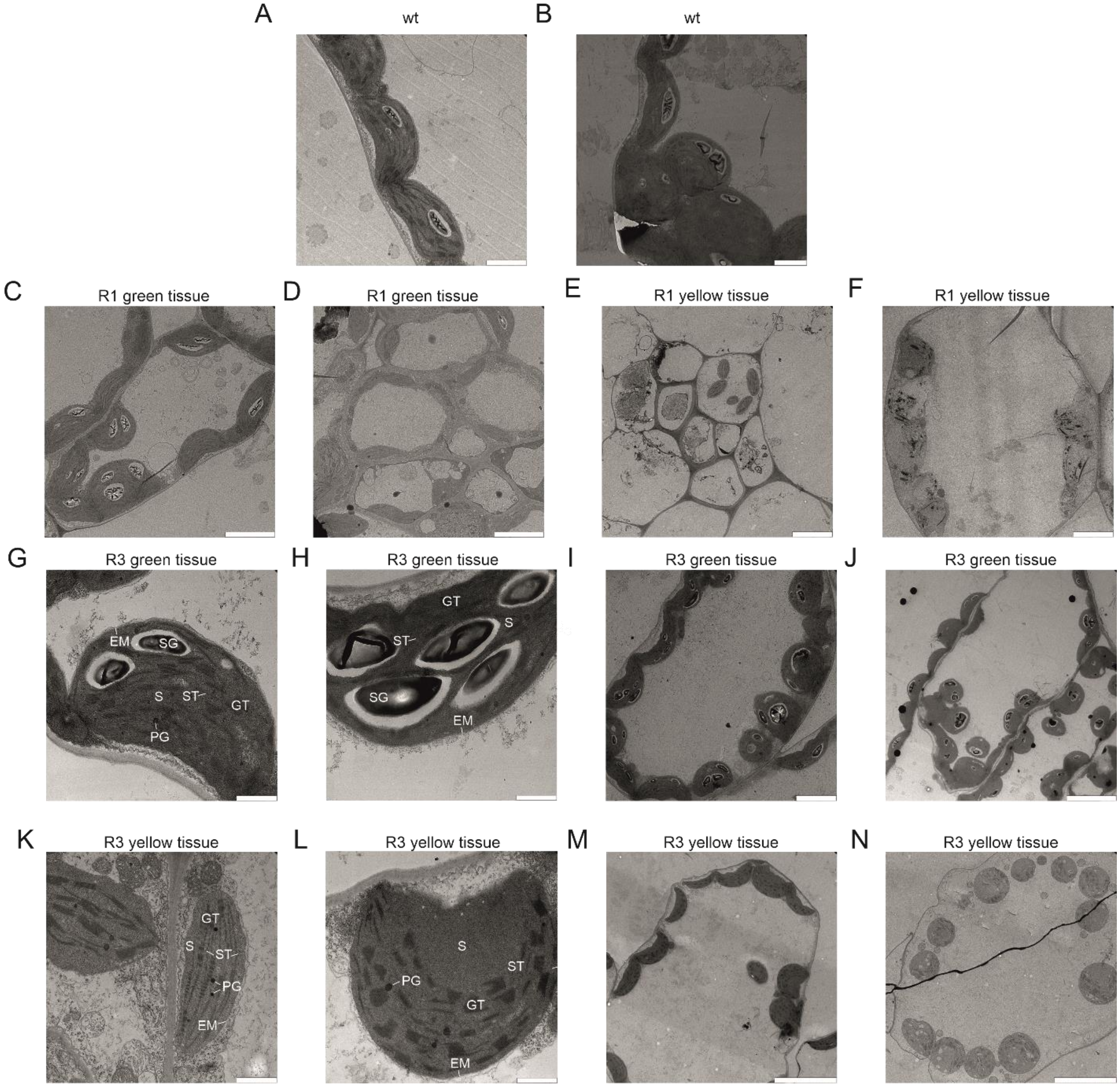

Supplementary Fig. S12. Transmission electron microscopy (TEM) images of tobacco cells from the *NtLCYB* RNAi line R1 and R3. A, B, wild-type chloroplasts. C, D, Chloroplasts in the green tissue of R1 cells chloroplast resembled similar shape and position to those in the wild type. E, F, Yellow tissue cells of R1 leaves have very few chloroplasts. Those remaining chloroplasts are observed more internally away from the cell contour or unstructured inside the envelope membranes. G -J, chloroplasts from line R3 in the green part of the leaves are similar to those of the wild type but with increased numbers and altered structure of starch granules. K-N, in the yellow parts of R3 leaves there are wild type-like chloroplasts and rounder chloroplasts, the organization of chloroplasts in the yellow parts exhibit round chloroplasts that are detached

from the cell wall, whereas other cells have mainly wild type-like chloroplasts. EM: envelope membranes, GT: grana thylakoids, PG: plastoglobules, S: stroma, SG: starch granule, ST: stroma thylakoids, wt: wild type. Scale bar: A and B: 2000 nm; C-F: 5000 nm. G, H, K and L: 1000 nm; I: 5000 nm; J, M and N 10000 nm.

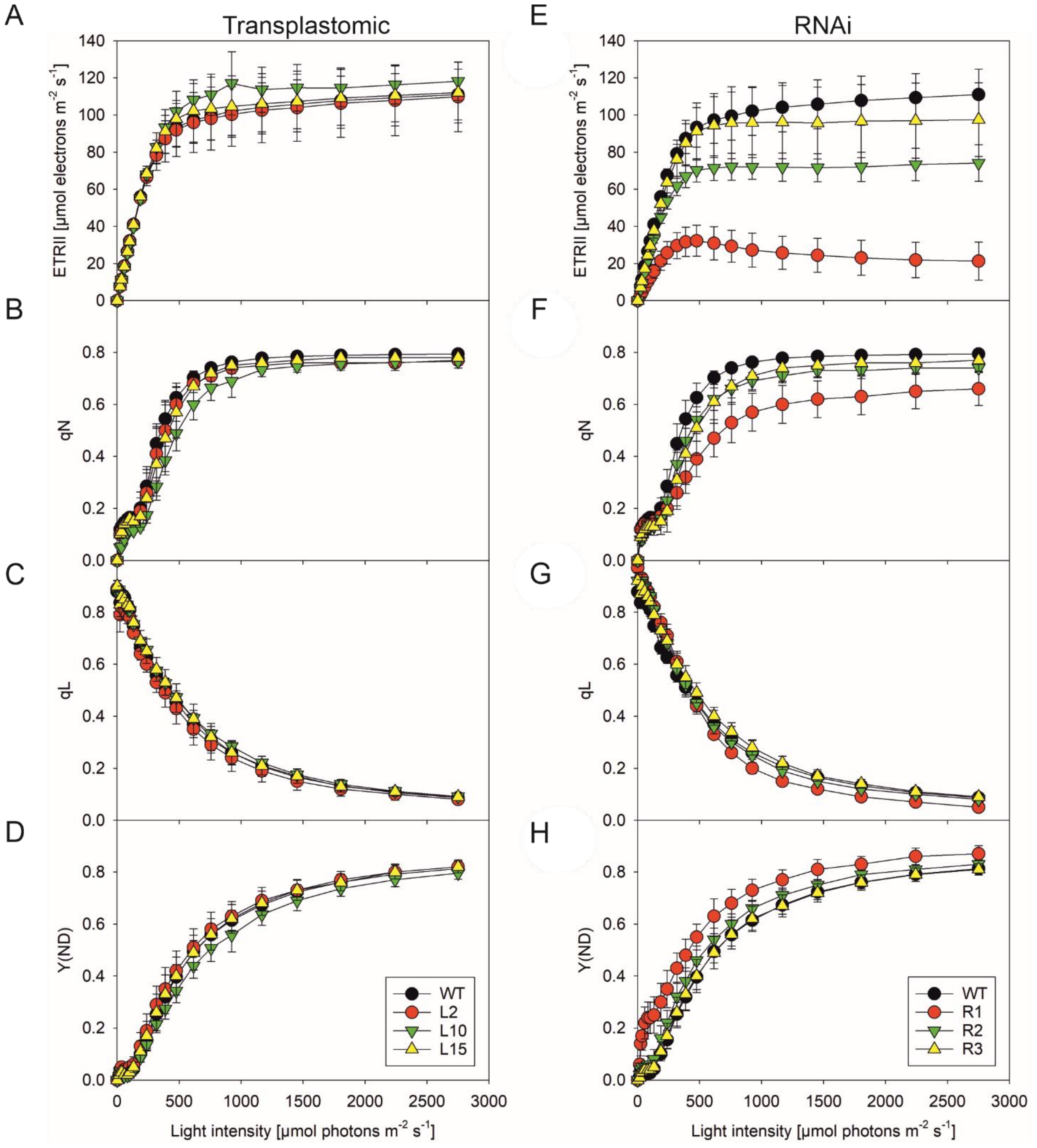

Fig. S13. Light response curves of photosynthetic parameters in transplastomic (A-D) and RNAi lines (E-H). A, E, Light response curves of linear electron transport were corrected for leaf absorptance. B, F, Light response curves of photoprotective non-photochemical quenching (qN). C, G, Light response curves of the redox state of the PSII acceptor side (qL). D, H, Light response curves of the donor-side limitation of PSI (Y(ND)).

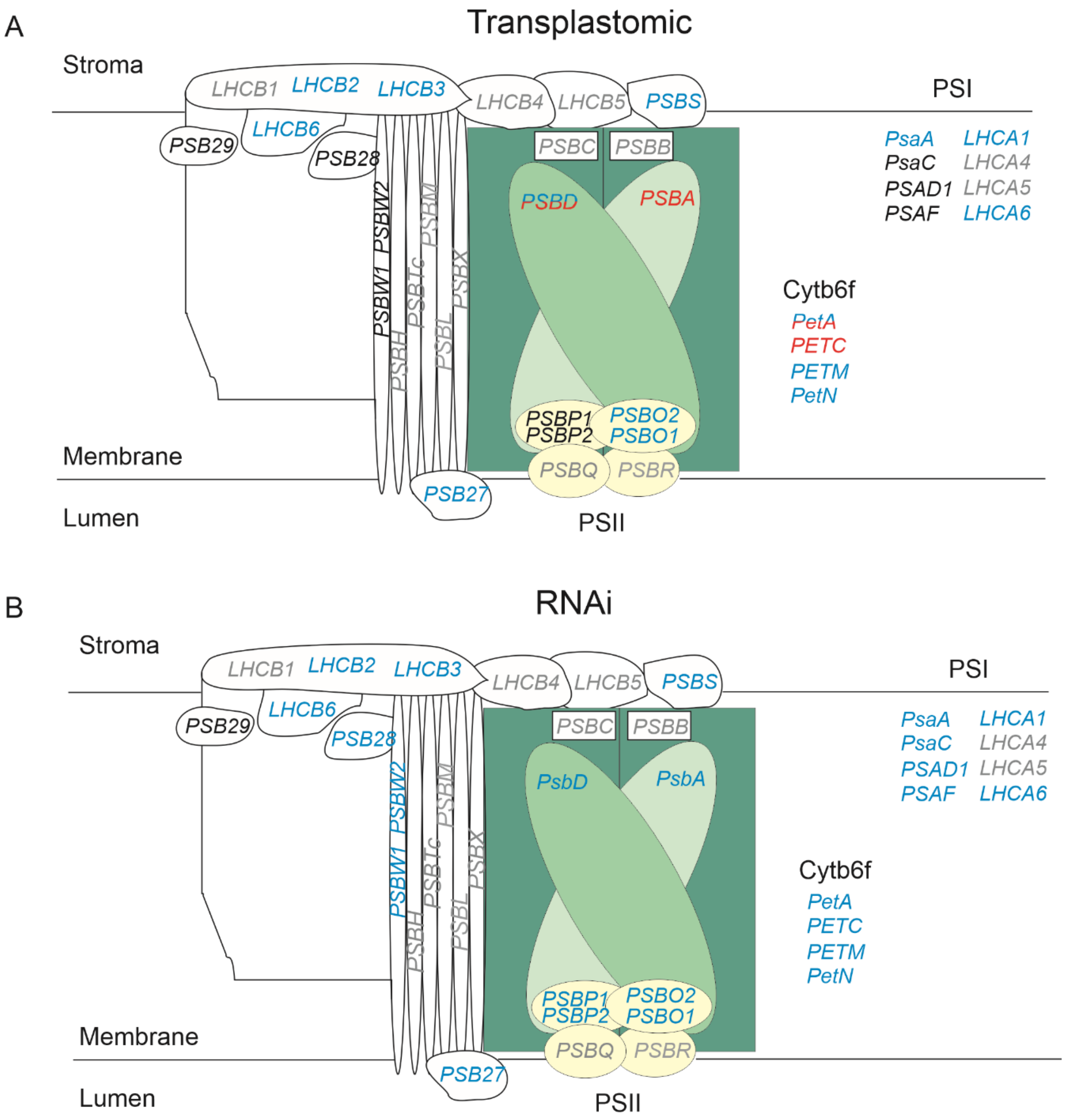

Fig. S14. Schematic model representing changes in gene expression of photosystem I, II and the cyt b_6f complex subunits in transplastomic and RNAi lines. A, changes in gene expression of transplastomic lines. B, changes in gene expression of RNAi lines. The model shows the increase (red) and decrease (blue) in gene expression of the photosystem I, II and the cyt b_6f complex subunits. Changes are marked if transcript accumulation was significantly changed in at least one line. Transcripts that are in one line up and in another

down are simultaneously marked in blue and red. Unchanged gene expression (black) and genes that were not measured (grey) are shown. PS: photosystem, Cyt b_6f: cytochrome b_6f complex.

Supplementary Tables

Table S1. Primers used for qPCR experiments.

Oligo name	Oligo sequence (5' to 3')	Reference
DcLCYB F	TTGACCTTCCTTTGTATGACCCGTC	Moreno et al., 2016
DcLCYB R	TCCTGCCTCAGAAACTTGTTGTGC	Moreno et al., 2016
NtPSY1 F	GGAACCAAGCTAATGACCCCAGAGAGA	Moreno et al., 2016
NtPSY1 R	GGCCGCCCACTGAAAATATCTTCC	Moreno et al., 2016
NtPSY2 F	TCAGAGATGTTGGAGAAGATGC	Moreno et al., 2016
NtPSY2 R	GCTTCAATCTCGTCCAATATCTTG	Moreno et al., 2016
NtLCYB F	CCGTGTTAAATTCCACCACGCCAA	Moreno et al., 2016
NtLCYB R	GAAGCCAGTTGCATCAAGCACCAC	Moreno et al., 2016
NtGGPPS F	GTATTGGGTTGTTGTTTCAAGTTGTGGAG	Moreno et al., 2016
NtGGPPS R	GCAATCAATGGAGCTGCTTTGTCTGGATC	Moreno et al., 2016
NtDXS1 F	GCCTTAGATGGACTTCTTGATGGCAAGT	Moreno et al., 2016
NtDXS1 R	TGTTAAACACTGTTGCTGCAATGTGAGAT	Moreno et al., 2016
NtDXS2 F	AGAGCATAACAAAGCAAATTGGACCTC	Moreno et al., 2016
NtDXS2 R	CTCCTCAAAAAGAGTTGAACAAGAAGCAC	Moreno et al., 2016
NtCHL F	ATCAAATATGGGTGCTTCTTCTTGGAGG	Moreno et al., 2016
NtCHL R	ATTATGTCAGGTGTAAGGGTGCCGAACA	Moreno et al., 2016
NtCPS F	ACACTAAAGCTGACATGGATACCAAAGG	Moreno et al., 2016
NtCPS R	CATAAGTGCAAAGGCAGTAGAAGATGGA	Moreno et al., 2016
NtKS F	TACTTACTACCGTGGTTGATGACTTCTTG	Moreno et al., 2016
NtKS R	CTTCATCTCCAATCTCACAAATAGTGCTT	Moreno et al., 2016
NtGA20ox1 F	TGTAGCACGAGAACTTCC	Moreno et al., 2016
NtGA20ox1 R	ACGGCATGCTTCACCAACA	Moreno et al., 2016
NtGA3ox1 F	CTTTACCAAAAACAGCCGACCCACG	Moreno et al., 2016
NtGA3ox1 R	CACCAATGGCTTTAGGAAAAACAGGACG	Moreno et al., 2016
NtNCED F	CTTTACCAAAAACAGCCGACCCACG	Moreno et al., 2016
NtNCED R	CACCAATGGCTTTAGGAAAAACAGGACG	Moreno et al., 2016
NtVDE F	ACTGAAAGAGTGCAGGTTAGAG	Kromdijk et al., 2016
NtVDE R	TCCGTTTCGTCAGGTCTATTG	Kromdijk et al., 2016
NtZEP F	CATGCCCGGATATCCTACAAA	Kromdijk et al., 2016
NtZEP R	CGGTATCTTCTGCCTTCGTTAT	Kromdijk et al., 2016
PSAA F	ATTCGTTCGCCGGAACCAGAAG	Armarego-Marriott et al., 2019
PSAA R	GCATGTAGGTTCCAGATCCAAGTG	Armarego-Marriott et al., 2019
PSAC F	GGATGTACTCAATGTGTCCGAGC	Armarego-Marriott et al., 2019
PSAC R	GGACAGGCGGATTCACATCTCT	Armarego-Marriott et al., 2019
PSAD1 F	GCGAGGAAAGAGCAGTGTTTAGC	Albus et al., 2012
PSAD1 R	CTTGACGGCCAGCGTTCACC	Albus et al., 2012
PSAF F	GATGGATTGCCACATTTGATAGTGAG	Hojka et al., 2014
PSAF R	ATCCAACCAGCAATGTACAAGAAGAG	Hojka et al., 2014
PSBA F	CAATTTTAGAGAGACGCGAAAGCG	Armarego-Marriott et al., 2019
PSBA R	GTAGGGATCATCAAAACACCAAACC	Armarego-Marriott et al., 2019

PSBD F	TGGTGCAAATACATTCCGTGCT	Armarego-Marriott et al., 2019
PSBD R	AGCGGTGACCATTGAATAAGTTTCT	Armarego-Marriott et al., 2019
PSBO1 F	AAACAGCTAGTGGCAAGCGGC	Albus et al., 2012
PSBO1 R	CAATGCAACAGCGTTGTCATAGCC	Albus et al., 2012
PSBO2 F	TCAAACAGCTCGTGGCATCTGG	Albus et al., 2012
PSBO2 R	CCCTTCCCTTTGGGTCAAGGAATG	Albus et al., 2012
PSBP1 F	ACAACGGGGATGGATTCAAGCTGC	Moreno et al., 2020
PSBP1 R	TGACCAGGGTACTCTACTTCCTTGT	Moreno et al., 2020
PSBP2 F	ACAACGGAGATGGATTCAAGTTGC	Moreno et al., 2020
PSBP2 R	ACCAGGGAACTCAACTTCTTTGC	Moreno et al., 2020
PSBS F	TGGCACAACTGGGAATTGCTTTC	Kromdijk et al., 2016
PSBS R	TGCCAAAGCTCCTTTCCCTGTG	Kromdijk et al., 2016
PSB27 F	CGGGCCTCATTCCGTGACATTTAC	Moreno et al., 2020
PSB27 R	CGGCCCAAAGCTAACATAATGGC	Moreno et al., 2020
PSB28 F	GGGGATATCACTGGATTCTACATG	Moreno et al., 2020
PSB28 R	AACCTGTCCCATTCTCGTGGAG	Moreno et al., 2020
PSB29 F	CAAGAAATCCTACCGATATGATCCC	Moreno et al., 2020
PSB29 R	GCTTTGAAGATGGCATCACGATCC	Moreno et al., 2020
PSBW1 F	CAGCAATGGCTCTTGTTGATGAG	Moreno et al., 2020
PSBW1 R	GTTGCTGAGACCAAAGGGAAGG	Moreno et al., 2020
PSBW2 F	GCAGCTATGGCTTTAGTGGATGAA	Moreno et al., 2020
PSBW2 R	GATTATTGCTCAAACCAAATGGAAGC	Moreno et al., 2020
PETA F	CCATTTTTGCACAGCAGGGTTATG	Armarego-Marriott et al., 2019
PETA R	ACCTGTTTCAGTTGCATATCATAAGG	Armarego-Marriott et al., 2019
PETC F	ATTTGCCCCTGCCATGGATCTC	Albus et al., 2012
PETC R	TGGGACAAACACCACCTTCCCA	Albus et al., 2012
PETM F	TGTCTTCAACCTGCAGTGTTGC	Hojka et al., 2014
PETM R	CAATCCGGAGGAGGACAAAAC	Hojka et al., 2014
PETN F	AGTAAGTCTTGCTTGGGCTGCT	Armarego-Marriott et al., 2019
PETN R	CCCACACTACGAGTGAAAGGGAA	Armarego-Marriott et al., 2019
CHLD F	AAGCTTCTGATGCACCCAGACC	Albus et al., 2012
CHLD R	AGCCACCTCGAGAATCTCATCC	Albus et al., 2012
GUN4 F	CAAGCTGACGAGGAAACTCGGC	Albus et al., 2012
GUN4 R	CTTCAGAGAAGAAAACATAGCCTCG	Albus et al., 2012
CHLM F	GAAGGACTTGGAGAGTTTGGATGG	Albus et al., 2012
CHLM R	CAGCCAATGACGCAAGATGAGC	Albus et al., 2012
CHL27 F	AGCACAGCCTCAGTTCCTCAATG	Albus et al., 2012
CHL27 R	AAGGCAGAAGAAGCGAGACCAC	Albus et al., 2012
PORC F	GGTCGTACGTGATCCAAGCCTTTC	Albus et al., 2012
PORC R	AACGAGGAGGAGGTGTTGTTCC	Albus et al., 2012
DVR F	GGTAAGTACAGTCCCTTATGCAATTC	Albus et al., 2012
DVR R	GGGCTTTCAATAGTAGAGGCAGC	Albus et al., 2012
CAO F	GCTAAAGGATGGACTGTCCCAAGC	Moreno et al., 2020
CAO R	TCCTTGAAGACCAGATGCAGGAG	Moreno et al., 2020

LHCA1 F	TAGCCATTGCTTTTGTAGAGCACC	Albus et al., 2012
LHCA1 R	GAGTAGCCCAATGGGTCAAAAGC	Albus et al., 2012
LHCA6 F	GGAGATCAAGAATGGTCGACTCGC	Albus et al., 2012
LHCA6 R	TGTCCAGGATCAGCAAGATGTGAC	Albus et al., 2012
LHCB2 F	GTTAAATTCGGTGAGGCAGTTTGG	Albus et al., 2012
LHCB2 R	AGGTAGTCAAGACCGCCTTCTG	Albus et al., 2012
LHCB3 F	TGGTTCAAAGCAGGAGCTCAAATC	Albus et al., 2012
LHCB3 R	GCATGCACAAGGTTAGGGTTGC	Albus et al., 2012
LHCB6 F	TGCACCTTTCTCTTTTGGCTCACT	Albus et al., 2012
LHCB6 R	CCTTTTGCTCTCAACCCACCCC	Albus et al., 2012
CHLH F	CCTCAGGAAAGACGGGAAGAAG	Albus et al., 2012
CHLH R	AGCCTCACAGTCTCAGACAACG	Albus et al., 2012
CHLI F	GTCCGTGACAAATTGAGGAGCGG	Albus et al., 2012
CHLI R	TGCCACACACCCTGTCCTCAG	Albus et al., 2012
PORAB F	CTCCTTTTCCCTCCATTCCAGAAG	Albus et al., 2012
PORAB R	AGGCTTGGATCACTTACAACCTG	Albus et al., 2012
CHLG F	GCTCAAACAGAATGGATGGATTGG	Albus et al., 2012
CHLG R	CCATAGCTCTGTCTCCTTCAATGC	Albus et al., 2012
ACT F	CCTCAGGTCCTTTTCCAACCA	Moreno et al., 2018
ACT R	GGATTCCGGCAGCTTCCATT	Moreno et al., 2018

* All the primers used in this study were previously published. Therefore, all of them were already tested with at least 3 different reference genes. For this reason, in this study we choose *ACTIN* as a reference gene.

Table S2. *In vitro* regeneration process for the generation of *DcLCYB1* transplastomic tobacco plants.

**Line**	**Spec^R/ Strep^R**	**Spec^R/ Strep^S**	**Obtained transplastomic lines**	**Analyzed lines by RFLP**
pJM36/*LCYB1*	2	20	1[a]	1
pJM37/*LCYB1*	23	18	23	6

*Spec: spectinomycin; Strep: streptomycin; R: resistant; S: sensitive. [a]: one transplastomic pJM36/*LCYB1* line dyed during the in vitro regeneration process.

Table S3. End-point measurement of plant height and flower number of 16-week old *NtLCYB* RNAi lines (n=3).

Line	plant height [cm]	flower number
R1	110.1 ± 5.3*	73 ± 22*
R2	127 ± 9.5	116 ± 5
R3	120.3 ± 7.6	175± 11**
wild type	122.9 ± 5.3	143± 18

Non-paired two-tailed Student's t-tests were performed to compare transgenic lines with the wild type: *: $p<0.05$; **: $p<0.01$.

Table S4. Chloroplast number per area in transplastomic *DcLCYB1* and *NtLCYB* RNAi lines (n=20 cells).

Line	Number of chloroplasts per 1000 μm^2
L2	22 ± 0.004*
L15	29 ± 0.006***
R1	8 ± 0.004***
R3	13 ± 0.004***
wild type	19 ± 0.004

Non-paired two-tailed Student's t-tests were performed to compare transgenic lines with the wild type: *: $p < 0.05$, ***: $p < 0.0001$.
